# Harnessing Membrane-Active Peptides for Selective Cancer Targeting: Phosphatidylserine Recognition by Tilapia Piscidin 4

**DOI:** 10.1021/jacsau.5c01524

**Published:** 2026-04-07

**Authors:** Wright K. Makambi, Soumita De, Dandan Li, Kinjal Mondal, Megan E. Mitchell, Navleen Kaur, Erik Watkins, Frank Heinrich, David P. Hoogerheide, Jeffery B. Klauda, Udo Rudloff, Myriam L. Cotten, Mihaela Mihailescu

**Affiliations:** † Institute for Bioscience and Biotechnology Research, Rockville, Maryland 20850, United States; ‡ 3421 National Cancer Institute, Bethesda, Maryland 20892, United States; § University of Maryland, College Park, Maryland 20742, United States; ∥ 10833 National Institute of Standards and Technology, Gaithersburg, Maryland 20899, United States; ⊥ Oak Ridge National Laboratory, Oak Ridge, Tennessee 37830, United States; # Department of Physics, 6612 Carnegie Mellon University, Pittsburgh, Pennsylvania 15213, United States; ∇ Oregon State University, Corvallis, Oregon 97331, United States; ■ National Institute of Standards and Technology, Gaithersburg, Maryland 20899, United States

**Keywords:** antimicrobial peptides (AMPs), anticancer peptides, biophysical characterization, cancer cells, lipid bilayers, membrane-active peptides, membrane asymmetry, molecular dynamics simulations, neutron diffraction, X-ray diffraction, neutron reflectometry, piscidins, tilapia piscidin 4 (TP4)

## Abstract

Membrane-active peptides (MAPs) have garnered significant attention as potential alternatives to conventional cancer therapies, which are frequently limited by severe side effects. Among them, antimicrobial peptides (AMPs) that leverage differences between the plasma membranes of cancer cells and healthy cells are particularly attractive. While several AMPs have demonstrated anticancer potency, structure–function relationship studies are lacking to explain the molecular basis of their selectivity and to help design improved analogs. Here, we contribute to filling this gap by investigating Nile tilapia piscidin 4 (TP4), an AMP with demonstrated activity against several solid organ cancers. First, we discover through biological assays that the anticancer activity of the peptide, which underscores a promising therapeutic window, is associated with increased plasma membrane permeability in cancer cells compared to normal cells and positively (negatively) correlated with enzymes that enrich (deplete) anionic PS in the outer leaflet. Next, we utilize a suite of complementary techniques on model membranes to investigate the interactions of TP4 with membranes, uncovering behaviors not previously observed in related AMPs. Circular dichroism experiments reveal that TP4 preferentially binds to zwitterionic phosphatidylcholine (PC) membranes enriched in anionic PS, while cholesterol markedly impairs binding. X-ray diffraction demonstrates that TP4 disrupts PC–PS membranes by inducing lipid segregation. Covering a range of biologically relevant peptide concentrations with neutron diffraction and reflectometry measurements in fluid bilayers and MD simulations, we unveil how TP4 and associated water gradually insert into the hydrocarbon region and cause convoluted membrane deformations to breach the membrane barriers. These studies highlight the pivotal role of the TP4 polyarginine tail in driving selective membrane binding and disruption on membranes enriched with the anionic lipid PS. Together, our results elucidate the molecular determinants underpinning the selective anticancer effects of TP4, providing a strategic framework for the rational design of advanced membrane-active therapeutics.

## Graphical abstract

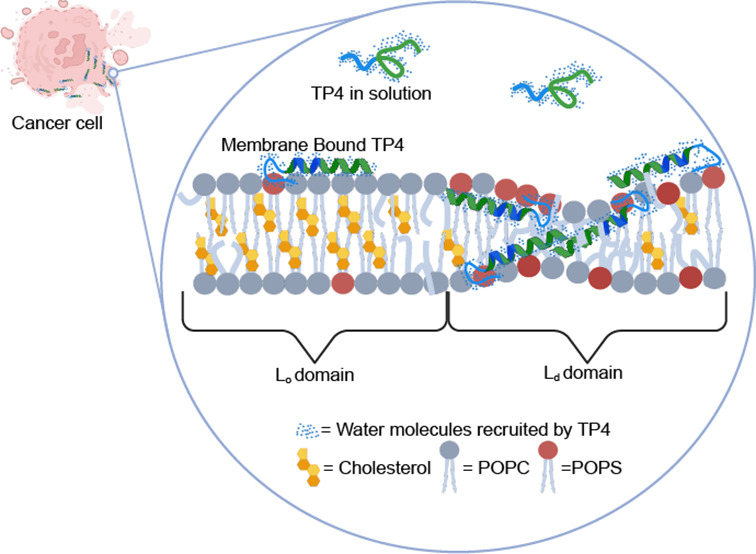

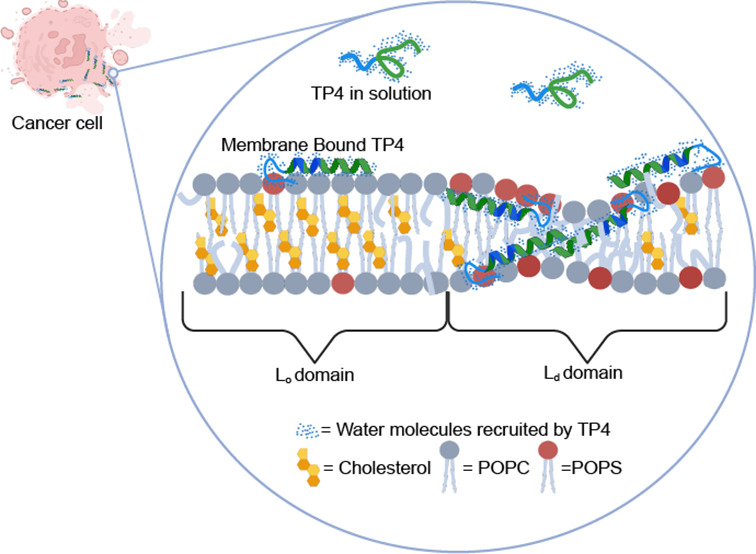

## Introduction

Cancer remains the second leading cause of death globally, with certain subtypes, such as lung, pancreatic, and brain cancers, carrying especially poor prognoses due to late-stage detection.^1^ Current treatment strategies primarily involve surgery and radiation therapy, with chemotherapy serving as the main intervention for stage IV cancer, the main driver of cancer-related mortality. Systemic cytotoxic chemotherapy, which exploits the increased division of cancer cells, typically affords short-term disease control. Indeed, its narrow therapeutic window is associated with frequent collateral damage to healthy tissues and significant side effects.^2,3^ Since the early 2000s, these limitations coupled with advances in decoding tumor genomes have propelled precision medicine, which leverage molecular differences between normal and malignant cells.^4,5^

While the selective targeting of surface receptors (e.g., estrogen,^6,7^ CD44,^8^ and HER2 ^9^) has led to approved therapies, the benefit of these strategies is often temporary, as cancer cells usually develop resistance to targeting these oncogenic drivers. Resistance to epitope-specific anticancer drugs can derive from mutations and epigenetic changes that alter the structure or expression of drug targets, as well as from the activation of redundant signaling pathways, which desensitize them to ligand-based drugs.^10,11^

In this research, we focus on the alternative strategy of targeting cancer cell membranes with cationic, amphipathic membrane-active peptides (MAPs). This approach has emerged from the understanding that cancer cell membranes display unique properties and biophysical features distinct from those of the plasma membrane of normal, nontransformed cells. For example, phosphatidylserine (PS), a negatively charged phospholipid that constitutes approximately 10–20% of the plasma membrane of mammalian cells^12–15^ and is typically restricted to the inner leaflet of healthy cell membranes, becomes exposed on the outer leaflet of many cancer cells.^12,16–18^ Indeed, similar to bacterial and yeast cells, cancer cells display negatively charged lipids on their outer leaflet to mimic the features of programmed cell death, and thereby evade innate and adaptive immune responses.^18^ Importantly, these alterations to biophysical properties create vulnerabilities that novel anticancer therapeutics could exploit.

Antimicrobial peptides (AMPs) are a class of naturally occurring MAPs that are extensively studied for their activity against microbial pathogens. Importantly, some AMPs, many of which are membrane active, also exhibit selective cytotoxicity toward cancer cells,^19–21^ likely due to the preferential interactions between their cationic surfaces and negatively charged membranes,^22^ making them compelling options as novel therapeutics. Among these, the piscidins, which belong to a family of histidine-rich AMPs derived from fish, were recently reported to exhibit anticancer properties.^23–26^ Notably, piscidin 1 (P1, FFHHIFRGIVHVGKTIHRLVTG), piscidin 3 (P3, FIHHIFRGIVHAGRSIGRFLTG), and tilapia piscidin 4 (TP4, FIHHIIGGLFSAGKAIHRLIRRRRR) exhibit cytotoxicity toward difficult-to-treat cancers, including nonsmall cell lung cancer,^27,28^ synovial sarcoma cells,^29^ or triple-negative breast cancer.^30^

While the structures of members of the AMP piscidin family are generally conserved, including multiple membrane-anchoring phenylalanines, numerous histidines, and a few cationic residues, several features distinguish them and could play a crucial role in their individual mechanisms of action. For instance, unlike other piscidins, TP4 has a unique polyarginine (R5) tail at the C-terminus, with our prior work demonstrating that the inclusion of the polyarginine tail results in enhanced activity against Gram-positive and Gram-negative bacteria, and greater membrane structure perturbations in membranes containing negatively charged lipids such as lipopolysaccharide (LPS) and 1-palmitoyl-2-oleoyl-*sn*-glycero-3-phospho-l-glycerol (POPG).^31^ This highlights the polyarginine motif as an essential feature in TP4’s enhanced selectivity for negatively charged membranes.

TP4 has demonstrated broad anticancer efficacy across different cell lines associated with solid organs. To date, there have been different mechanisms of action, including the induction of necrosis in glioblastoma via ROS-mediated DNA damage^32^ or the disruption of calcium homeostasis in triple-negative breast cancer and synovial sarcoma cells.^29,30^ Recent reports have also suggested that apoptosis may play a role in TP4-induced cell death,^33,34^ with several studies describing antimitochondrial functions of TP4 and the induction of the intrinsic pathway of programmed cell death as the primary mechanism against cancer cells.^28,30,32–34^ Despite this progress, a detailed biophysical characterization of TP4-induced changes within lipid bilayers, including how TP4 inserts into the plasma membrane and reaches intracellular targets, is still missing.

In this study, we hypothesize that enrichment of PS present in the outer leaflet of cancer cells membranes helps confer selective susceptibility to TP4. We used biological assays to verify this hypothesis and identified a promising therapeutic window for TP4. We deployed a series of complementary biophysical methods on model membranes to elucidate the mechanism underlying the preference of the peptide for anionic lipids and its contribution to the membrane-disruptive behavior. Our comprehensive study discovers three key results: (1) bilayer charge and fluidity are key determinants of the membrane binding specificity and disruptive effects of TP4; (2) TP4 inserts deeply into the membrane, inducing dramatic structural disruptions driven by the influx of water into the bilayer and the blurring of boundaries between hydrophobic and polar regions; (3) and the polyarginine tail of TP4 is essential to its enhanced membrane activity. Overall, our findings elucidate the basis of the selective anticancer activity of TP4 and highlight arginine-rich peptides as promising candidates for improved cancer therapies.

## Methods

### Peptides and Reagents

The following peptides, TP4, P1, P3, and P1-R5 (FFHHIFRGIVHVGKTIHRLVTGRRRRR) were chemically synthesized in their carboxylamidated form at the University of Texas Southwestern Medical Center and Tufts University. Once received, they were purified and lyophilized, as previously reported.^35^ The peptides were diluted in HCl to exchange trifluoroacetate ions with chloride ions and dialyzed into nanopure water to remove excess salts, resulting in peptide purity of 98%.^35,36^ After reconstituting the peptides in nanopure water, their molar concentrations were determined by amino acid analysis performed at the Protein Chemistry Center at Texas A&M. TP4 and TP4-noR5 (FIHHIIGGLFSAGKAIHRLI) used in NR measurements were produced by Biomatik (Kitchener, Ontario, Canada) at a purity >95%, and processed as above.

### Cell Lines

Human pancreatic cancer cell lines PANC-1, PSN-1, MIA PaCa-2, HeLa, fibroblast GM05659, HEK293, RAW 264.7, and normal pancreatic ductal epithelial cells (HPNE; cat #CRL-4023) were obtained from ATCC (Manassas, VA), PC3-M from Cytion (cat. #305061; Sioux Falls, SD), PK-8 and PK-59 from the RIKEN BRC Cell Bank (Japan), and YAPC from the Leibniz Institute DSMZ-German Collection of Microorganisms and Cell. Cells were maintained according to the supplier’s instructions or in RPMI-1640 medium supplemented with 10% (v/v) fetal bovine serum (FBS) and incubated at 37 °C in a 5.0% CO_2_ atmosphere. In accordance with American Association for Cancer Research (AACR) practices, cell lines were confirmed by SNP genotyping using Illumina MiSeq sequencing and tested for the presence of mycoplasma using the MycoAlert Mycoplasma Detection Kit from Lonza. Gene expression profiling of pancreas cancer cell lines was done using the HumanHT-12 v4 Expression BeadChip (Illumina Inc., San Diego, CA) described previously.^37^

### Cancer Cell Viability Assay

The inhibitory concentration at 50% reduction in viability (IC_50_) was determined using the CellTiter-Glo assay (Promega, Madison, WI). Cell lines were plated at a cell density of 5000 cells/well and allowed to adhere overnight. Serial dilutions of peptides were prepared in water in a separate 96-well plate and added to the previously plated cells to a final concentration of 0.01 to 30 μmol/L peptide. Cells were incubated with added peptides at 37 °C for 24 h, following which cell growth was assessed using the CellTiter-Glo assay with levels of untreated cells normalized to 100%. Data analysis was performed using GraphPad Prism version 10 software. Dose–response curves were created using a four-parameter equation fitting technique, and the 95% confidence intervals for each sample were provided. IC_50_ values were listed as not determined (ND) if no inflection point was reached for observed values. Statistical analysis comparing P1 and P1R5 was performed using a paired *t* test, assuming a normal distribution, with a false-negative rate of 0.01.

### Dual Calcein AM/Ethidium Homodimer-1 Staining

Changes in membrane permeability and cell viability were assessed using the Live/Dead Viability Cytotoxicity Kit (ThermoFisher Scientific, Grand Island, NY). Cells were seeded onto glass-bottom 96-well plates and treated with vehicle and different concentrations of TP4 for 24 h. After drug treatment, a 100 μL mixture of 2 μmol/L calcein-AM and 4 μM Ethidium Homodimer-1 (EthD-1) was added and incubated for 1 h. Images were taken at 20× magnification on a Zeiss LSM880 Airyscan confocal microscope (Zeiss). Three separate images for each treatment group containing about 50 cells from 3 randomly selected fields across the three technical replicates were analyzed using ImageJ software. The number of automatically counted red and green fluorescent objects was normalized to the total number of fluorescent objects. For relative comparison, the fluorescence ratio for vehicle-treated cells was normalized to 100%, and the percentages of EthD-1 and calcein-positive cells were calculated. The dose−response curve was created using a four-parameter equation fitting technique, and the 95% confidence intervals for each sample were provided. IC_50_ values were listed as not determined (ND) if no inflection point was reached for observed values.

### Mitochondrial Function

Mitochondrial activity was assessed using MitoTracker Red FM (ThermoFisher Scientific, Grand Island, NY). Cells were plated at a concentration of 3000–5000 cells/well in 100 μL media, treated for 24 h with vehicle and different concentrations of TP4 in triplicate. After treatment, cells were stained with MitoTracker Red FM according to the manufacturer’s protocol, and fluorescence was quantified using an Agilent BioTek Synergy H1 multimode reader.

### Liposome Preparation

Small unilamellar vesicles (SUVs) were prepared to mimic the outer membrane of healthy and cancer cells. Lipids, 1-palmitoyl-2-oleoyl-*sn*-glycero-3-phosphocholine (POPC), 1-palmitoyl-2-oleoyl-*sn*-glycero-3-phospho-l-serine (POPS), 1-pentadecanoyl-2-oleoyl­(d7)-*sn*-glycero-3-phosphocholine (d7-PC) and cholesterol (bovine wool, >98%) were obtained from Avanti Polar Lipids (Alabaster, AL) as a powder. Before use, lipids were reconstituted with 1 mL of chloroform and mixed to the desired lipid composition. Chloroform was then removed under a stream of nitrogen for 15 min and further under a vacuum for 30 min. Lipids were then hydrated in water or buffer above their transition temperature and sonicated using a bath sonicator.

### Circular Dichroism Spectroscopy

CD spectroscopy was performed using a Chirascan Spectrometer (Applied Photophysics, Charlotte, NC), located at the Institute for Bioscience and Biotechnology Research (IBBR) in Rockville, Maryland. Experiments were conducted using an automatic titrator to incrementally add 200 μL of 3 mmol/L SUVs composed of POPC, POPC/POPS (5:1 molar ratio), POPC/cholesterol (2:1 molar ratio), or POPC/POPS/cholesterol (5:1:2.5 molar ratio) in 5 mmol/L sodium phosphate buffer (NaPB) containing 5 mmol/L NaCl at pH 7. The SUVs were titrated into a cuvette containing 300 μL of 20 μmol/L TP4 or buffer alone (for background subtraction) in 20 steps of 10 μL each, enabling a range of peptide-to-lipid (P/L) molar ratios from 1:5 to 1:100. Following each addition, the sample was stirred for 2 min and equilibrated for 2 min before measurement. CD spectra were acquired at 25 °C, over a wavelength range of 200–260 nm, with a spectral bandwidth of 1 nm and a data acquisition time of 3–5 s per point. The raw data, recorded in millidegrees, were first corrected for dilution effects and baseline drift. Background spectra were then subtracted from the peptide spectra, after which the data were converted to mean residue ellipticity (MRE) and normalized to the MRE of the peptide in buffer on the day of measurement to obtain the normalized MRE (nMRE).

To quantify the binding affinity and stoichiometry of TP4 interaction with lipid membranes, the dissociation constant (*K*_D_) and the number of lipids bound per peptide molecule (*n*) were determined by fitting the experimental data to a modified Langmuir binding model using a Markov Chain Monte Carlo sampling method. In this model, the fitted parameters include *K*_D_, *n*, and the maximum normalized MRE (nMRE_max_). The relationship used is as follows:

$$C_{\mathrm{Lt}}=nf_{b}\left[1+\frac{K_{D}}{C_{\mathrm{pt}}\left(1-f_{b}\right)}\right]C_{\mathrm{pt}}$$

Where *C*_Lt_ is the total lipid concentration (free and bound to peptide), *f*_b_ is the fraction of peptide bound, and *C*_pt_ is the total peptide concentration in the system (free and bound to SUVs). The fraction of peptide bound is linearly related to the nMRE by the following equation:

$$f_{b}=\frac{\mathrm{nMRE}-{\mathrm{nMRE}}_{\min}}{{\mathrm{nMRE}}_{\max}-{\mathrm{nMRE}}_{\min}}$$

Where nMRE is the measured normalized MRE, nMRE_min_ is the minimum normalized MRE (set to 1), and nMRE_max_ is the saturation value (free parameter). A complete derivation is provided in the Supporting Information.

The CD binding curves were fitted using a DREAM (Differential Evolution Adaptive Metropolis) code in MATLAB that performs Markov chain Monte Carlo (MCMC) simulations to explore the parameter space. The most probable parameter values were determined by drawing from a posterior distribution, which combines a Gaussian noise and prior constraints. The resulting posterior distributions allow estimation of the median values as well as uncertainties (e.g., 68% confidence intervals) for each parameter and are reported in Table 3.

### X-ray Diffraction

XRD measurements were performed using a 3 kW Rigaku SmartLab diffractometer located at the IBBR. XRD patterns were collected in θ–2θ reflection mode, where the incidence and detection angles were equal, probing structural features along the membrane normal (*z*-axis). Repeat spacings (d) and their associated uncertainties were calculated from the average of Bragg peak positions plotted against their corresponding diffraction orders. Bragg reflections were identified at positions where the momentum transfer *Q*_*z*_ = 4πλ^–1^sin­(θ) matched the condition *Q*_*z*_ = 2π*hd* ^–1^, where *h* is the diffraction index, and λ is the photon wavelength (1.54 Å).

Oriented multilayers (lamellar) samples of lipid with and without peptide were prepared as previously described.^38^ In short, lipid stacks were obtained by depositing 400 μL of 2 mg SUVs, with or without TP4 at a molar P/L of 1:25, onto clean substrates. The samples were left to dry overnight under ambient conditions, allowing slow evaporation of bulk water. This method yielded lamellar samples composed of approximately 1000 to 2000 bilayers, with peptides populating both sides of the bilayers through vesicle fusion and molecular exchanges. Before performing the diffraction experiments, the samples were equilibrated in a humidity-controlled chamber at either 86, 93, or 97% relative humidity (RH) and room temperature for several hours to ensure uniform hydration and ordering, and measurements were carried out at 25 °C.

### Neutron Reflectometry

NR experiments were performed on the LIQREF horizontal reflectometer at the Spallation Neutron Source at Oak Ridge National Laboratory. Liquid flow cells consisted of a silicon backing wafer and a sample wafer (each 5 mm thick and 2 in. in diameter) separated by a nominally thin Viton gasket, forming a reservoir with an estimated volume of 0.22 mL. Holes were drilled in the backing wafer for the inlet and outlet. Sample wafers were single-crystal silicon with a native oxide surface. A polychromatic beam of neutrons impinged on the interface between the surface of the sample wafer and the liquid in the sample cell reservoir. Each measurement covered a range in scattering wavevector *Q* = 4πλ^–1^sin­(θ) from 0.010 to 0.28 Å^–1^, binned to maintain a constant bin width equal to 1.0% of the bin center.

Lipid bilayers were formed via vesicle fusion. Lipids in chloroform were stored at −80 °C prior to use, mixed in the molar ratio 1,2-dioleoyl-*sn*-glycero-3-phosphocholine (DOPC)/1,2-dioleoyl-*sn*-glycero-3-phosphoserine (DOPS) (3:1 molar ratio), and dried under vacuum overnight. Vesicles were formed by reconstituting the dried films in a 1 mol/L potassium chloride solution and sonicating until translucent. Vesicles were introduced into the flow cell and allowed to incubate for 1.5 h, then flushed with pure water followed by buffer. Buffers were 10 mmol/L Tris pH 7.4 in either H_2_O or deuterium oxide (heavy water, D_2_O). After bilayer formation, subsequent buffer exchanges were performed manually or via syringe pump. For peptide additions, at least 4 mL of 3 μmol/L peptide dissolved in buffer was injected (to avoid depletion of the peptide solution) over the course of 10 min.

Four measurements were performed on each bilayer, one in each D_2_O and H_2_O before the peptide was added, and the same two contrasts thereafter. NR data were analyzed using the composition space modeling described previously.^39^

### Neutron Diffraction

Neutron diffraction (ND) measurements on lamellar samples were acquired with the CGD-MAGIK instrument at the NIST Center for Neutron Research (NCNR). Monochromatic, cold neutrons of wavelength λ = 5 Å and a wavelength spread Δ λ/λ = 1% were diffracted by the samples and counted with a pencil-type ^3^He gas-filled detector. Diffraction from (*h*,0,0) set of planes of the aligned multilayers, prepared as above for XRD, was recorded and used to determine the one-dimensional scattering-length density (SLD) profiles of the bilayer along the *z*-axis. The data sets were processed and analyzed as described before.^40,41^ In short, bilayer structure factors were obtained as the square roots of the integrated Bragg peaks, corrected for background, absorption and extinction, and their phases determined by deuterium contrast, using H_2_O/^2^H_2_O exchange.^40,42,43^ Up to five orders of diffraction (*h* = 1–5) were observed for each of the measured samples in this study (Figure S9). Tables with structure factors can be found in Supporting Information (Tables S2). The contrasts between deuterium-containing and natural abundance samples arising from the higher neutron scattering length of deuterium (*b* ^2^ *H* = 6.67.10^–5^ Å) with respect to hydrogen (*bH* = −3.74.10^–5^ Å) were used to parse out the specific profiles of the deuterated regions, by deuterium difference.^43^ Here, deuterated lipids in the acyl chains (d7-PC) and in the water of hydration (^2^H_2_O) were used, in combination, for amplitude calibration of the SLD profile and quantification of the amount of bound water to the bilayers.^41,42,44^ Repeat distances, *d* and their uncertainties were determined by a linear fit of the Bragg peak position versus diffraction order. Deuterium peak shapes were analyzed using Gaussian models and Levenberg–Marquardt nonlinear least-squares fitting procedures, with χ^2^ weighted by the uncertainties (i.e., standard deviations, SD) in the measured data due to counting statistic. Fit parameter confidence intervals were determined by a Monte Carlo resampling technique,^42,45^ where a large number (*n* = 100) of statistically independent sets of mock structure factors values (normally distributed within ± 1 SD) were tested, thus producing one set of fit parameters for each iteration. Means and SDs of the fit parameters were calculated from these sets.

### Molecular Dynamics Simulations

Molecular dynamics (MD) simulations were implemented utilizing the Expanse SDSC cluster, through the University of Maryland cluster. Model membranes consisting of POPC/POPS (5:1 molar ratio) were set up around the TP4 molecules using a Highly Mimetic Mobile Membrane (HMMM) builder in CHARMM-GUI as previously described.^46^ In these models, the lipid tails are represented as organic solvents (1,1-dichloroethane-DCLE) to enhance sampling of peptide-membrane structures, without compromising atomic detail.

For these simulations, the system utilized TP4 (Protein Data Bank ID: 5H2S)^47^ and POPC/POPS (5:1 molar ratio) lipids at a P/L of 1:40. Four TP4 molecules were positioned parallel to the membrane plane, 5 Å from the bilayer surface. The membrane was constructed with equal numbers of lipids in the upper and lower leaflets. A TIP3P water box was added, containing equal numbers of water molecules above and below the bilayer and extending 5 Å beyond the peptide surfaces. The system was neutralized using 5 mmol/L NaCl ions and maintained.

In CHARMM-GUI, force-field parameters were assigned using CHARMM36 for lipids and CHARMM36m for the peptide.^48,49^ System minimization and equilibration followed the standard six-step CHARMM-GUI protocol. Minimization was performed using the steepest descent algorithm. Input files were generated for GROMACS to run equilibration under the NVT ensemble (constant number of particles, volume, and temperature = 303.15 K) and production under the NPAT ensemble (constant number of particles, pressure = 1 bar with semi-isotropic coupling, temperature = 303.15 K, and fixed bilayer area).^50,51^ The LINCS algorithm was applied throughout to constrain bonds involving hydrogen atoms. Flat-bottom restraints were also applied to confine the peptide within 6 Å of the box center. Production simulations were run for 500 ns.

For comparison with experimental data, the final frame from the HMMM trajectory was converted to an all-atom representation of the lipid tails, and an additional 100 ns simulation was performed. Electron density profiles at the peptide–membrane interface were calculated using the gmx density function in GROMACS, and radial distribution functions of peptide-associated water molecules were obtained with MD analysis.^52^

## Results

### TP4 Shows Stronger Anticancer Activity than Other Piscidin Homologues across a Panel of Pancreatic Cancer Cell Lines

To assess the potency of representative piscidins against pancreatic cancer cell lines, we determined their IC_50_ values. TP4, P1, and P3, including their chelated isoforms, were selected based on their previously reported anticancer activity.^27–30^ To investigate how the polyarginine tail impacts the activity of P1, we evaluated P1R5, a P1 variant with a polyarginine tail appended to its C-terminus. Several prior reports, including work from our group, have suggested disruption of mitochondrial membranes and impairment of mitochondrial respiration as the primary cytotoxic mechanisms of piscidins.^29,30,32,53^ To examine whether different metabolic dependencies—including different dependencies on mitochondrial metabolism—correlate with the activity profile of piscidin peptides, we selected cell lines with lipogenic and nonlipogenic metabolic phenotypes for these studies. Lipogenic cell lines rely on mitochondrial perspiration, including fatty acid metabolism and oxidative phosphorylation, and are more sensitive to inhibition of mitochondrial function.^54^ Cell viability was measured across a range of peptide concentrations (Figure 1), and IC_50_ values, which were derived as described in the Methods section, are summarized in Table 1.

**Figure 1:**
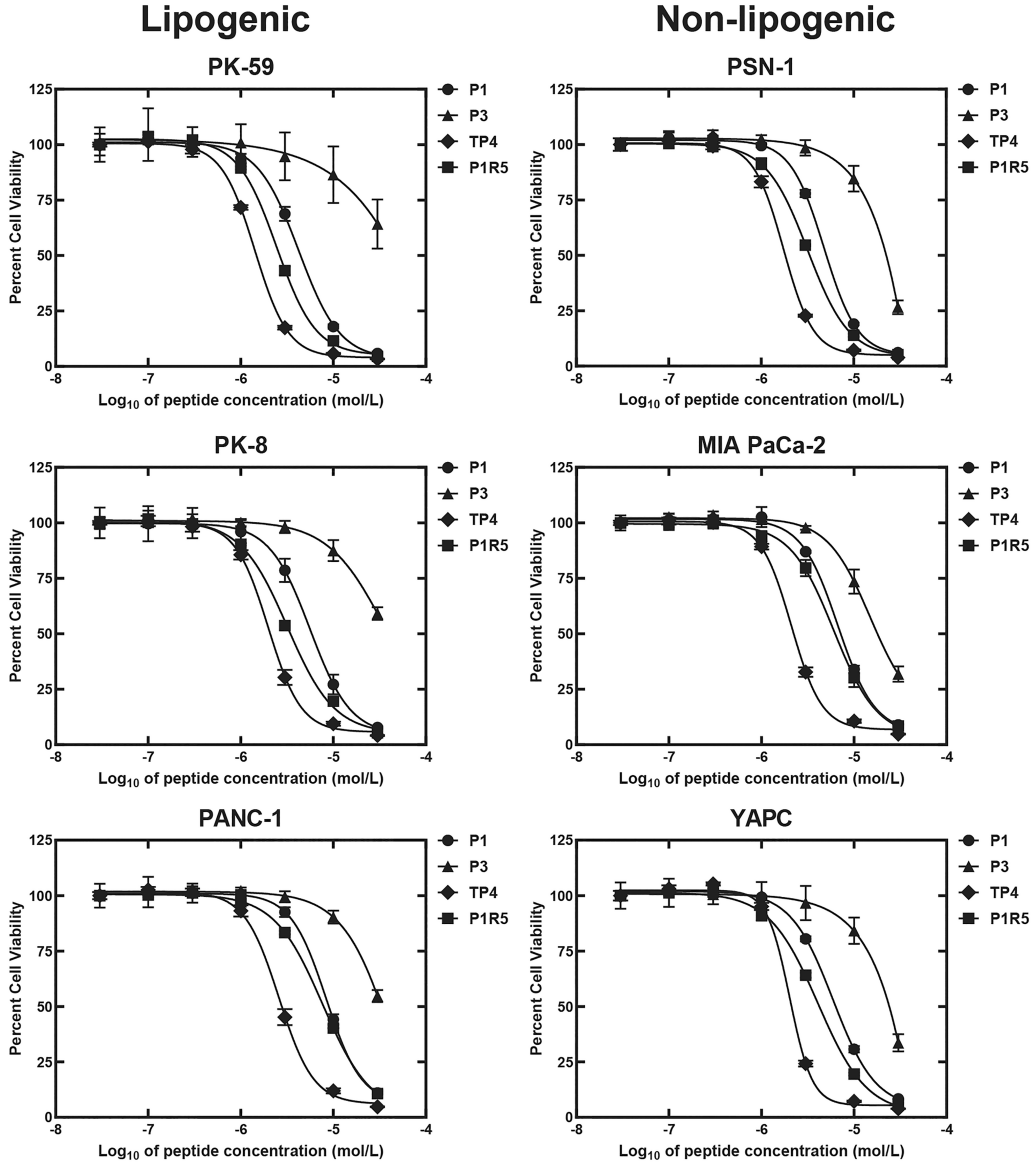
Anticancer activity of piscidin peptides against lipogenic and nonlipogenic pancreatic cancer cells. After peptides (P1, P3, TP4, or P1R5) were mixed with cells to the final concentrations indicated, the mixture was incubated at 37 °C for 24 h. Cell growth was assessed using the CellTiter-Glo assay, with levels of untreated cells normalized to 100%. Error bars represent the standard deviation for *n* = 3 replicates. Figures including peptides chelated with copper can be found in Figure S1 and IC_50_ are reported in Table S3. Controls for determining lipogenic and nonlipogenic cells are included in Figure S2.

**Table 1:** IC_50_ Values for All Tested Peptides, against Lipogenic and Non-Lipogenic Pancreatic Cancer Cell Lines^a^

IC_50_ (μmol/L)	Cell line	P1	P1R5	TP4	P3
Lipogenic	PK-59	4.23 (3.83–4.71)	2.45 (2.32–2.57)	1.42 (1.38–1.47)	ND^b^
	PK-8	5.60 (4.58–6.95)	3.19 (2.72–3.86)	1.97 (1.86–2.10)	36.4 (ND)^b^
	PANC-1	8.37 (7.42–9.59)	7.66 (7.10–8.34)	2.59 (2.44–2.75)	50.1 (ND)^b^
Nonlipogenic	PSN-1	4.77 (4.51–5.04)	3.17 (2.99–3.38)	1.74 (1.66–1.83)	ND^b^
	MIA PaCa-2	6.69 (6.14–7.27)	6.03 (5.18–7.12)	2.10 (2.00–2.20)	14.9 (11.18–35.51)
	YAPC	5.92 (5.56–6.32)	4.03 (3.72–4.40)	2.02 (1.89–2.17)	ND^b^

a IC_50_ are reported with 95% confidence intervals. *Q*-values comparing P1 and P1R5 can be found in Table S4.

b ND: not determined.

The viability assay revealed distinct activity profiles for each piscidin. First, for all piscidins, there was no clear preference for cells with the lipogenic phenotype, cell lines with greater dependency on mitochondrial function (Table S5). These findings are in line with TP4-treated PSN1 pancreatic cancer cells evaluated through the MitoTracker assay. The latter measures the retention of a cell-permeable dye in the mitochondria, a property dependent on mitochondrial mass and function (Figure S3). At concentrations where TP4 significantly decreased cell viability, the corresponding MitoTracker response were minimally changed compared to vehicle control. Together, these findings indicate that the primary mechanism of action for TP4, and possibly the other picidins, appears to be independent of direct mitochondrial targeting, and additional mechanisms are involved in the activity of TP4.

Next, when comparing P1 and P1R5, the addition of a polyarginine tail produced modest improvements in potency, with IC_50_ values shifting from 4.23–8.37 μmol/L (P1) to 2.45–7.66 μmol/L (P1R5) (Table S4). Given that P1 inserts from the C-terminus to exert its effect^53,55^ and the polyarginine tail was added to that end, P1’s altered potency after addition of the polyarginine tail might be influenced by a change in its mechanism of action.

Among the peptides tested, P3 was clearly the least active across all cancer cell lines, with IC_50_ ranging from 14.9–70.2 μmol/L, whereas TP4 was the most potent with IC_50_ values between 1.42–2.59 μmol/L. Notably, P3, which inserts in the membrane via its N-terminus, binds copper at its Amino-Terminal Copper and Nickel (ATCUN) binding motif.^53,55^ Consistent with prior studies, copper chelation enhances the potency of all tested peptides, except for TP4, which was largely unaffected despite containing a similar ATCUN binding motif as P3 (Figure S1, Table S3). Notably, even in their chelated forms, the activity of the other peptide did not surpass that of TP4. Together, these findings suggest that TP4 may act through additional mechanisms compared to the other piscidins. Overall, TP4 as the peptide with the top cell-based activity, was selected for subsequent experiments.

### TP4 is More Active against Cancer Cells than Healthy Cells

Next, as the most active AMP, we studied the selectivity of TP4 against nonmalignant cells. Previous reports, which primarily used fibroblasts as control, have reported variable tumoral selectivity, with selectivity indices of less than 2-fold between normal and colorectal cancer cells to greater than 10-fold for breast cancer.^33,56^ To limit bias in the plasma lipidome due to different lineages and tissue origins when contrasting cancer versus normal cells, we first measured the activity of TP4 on pancreatic cancer cells (PANC1 and PSN1) and pancreatic normal ductal epithelial cells (Human Pancreatic Nestin-Expressing, HPNE); the latter are nontumorigenic human pancreatic ductal cells, which transform via pancreatic intraepithelial neoplasia (PanIN) precursor lesions into pancreatic ductal adenocarcinoma (PDAC). TP4 suppressed growth more effectively in cancer cells than nonmalignant cells, with median IC_50_ values in normal pancreatic ductal HPNE cells 10-fold higher (Figure 2, Table 2). Next, we expanded our screening to include embryonic kidney cells (HEK293), multiple patient-derived fibroblast lines, and nonpancreatic cancer lines—cervical (HeLa) and prostate (P3CM) carcinoma (Figure S4, Table S6). Across these diverse lines, we observed a therapeutic window consistent with our findings in the pancreatic models. While these results suggest a broad selectivity for the malignant phenotype, we acknowledge that differences in tissue origin and nonmatching histologies may also contribute to the observed variations in sensitivity.

**Figure 2:**
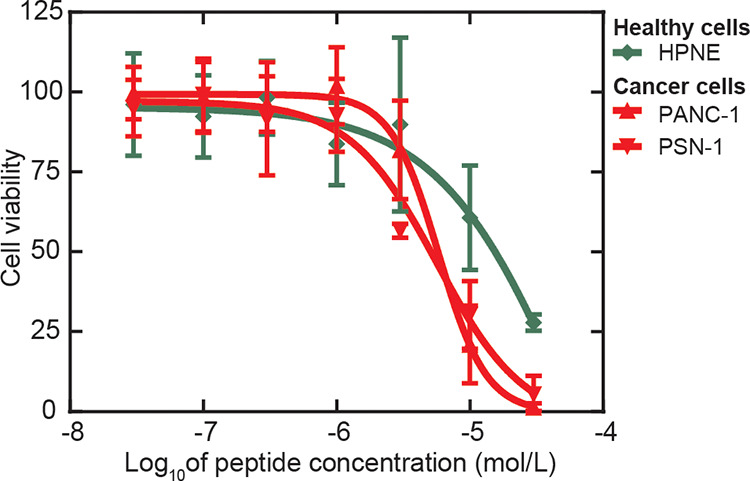
Anticancer activity of TP4 against healthy and cancer pancreatic cell lines. Cancer cells (PANC-1 and PSN-1) and pancreatic ductal epithelial cells (HPNE) were exposed to TP4 at the indicated concentrations. Mixtures were incubated at 37 °C for 24 h. Following incubation, cell growth was assessed using the CellTiter-Glo assay with levels of untreated cells normalized to 100%. Error bars represent the standard deviation for *n* = 2 replicates.

**Table 2:** IC_50_ Values for TP4 against Cancer Cells (PANC-1 and PSN-1) and Non-Malignant Epithelial Cells^a^

Cell line	PANC-1	PSN-1	HPNE
IC_50_ (μmol/L)	5.80 (4.77–6.97)	5.66 (3.31–16.8)	57.91 (ND)^b^

a IC_50_ are reported with 95% confidence intervals.

b ND: not determined.

### TP4 Induces Membrane Permeability in Cancer Cells

To connect TP4 cytotoxicity to alteration in plasma membrane integrity, we employed a dual Calcein AM/Ethidium Homodimer-1 (EthD-1) staining assay. Upon loss of membrane integrity, the impermeable red-fluorescent EthD-1 can enter the cells and bind to DNA in the nucleus. The cell-permeable calcein AM, an ester, is hydrolyzed in the cytoplasm to the green-fluorescent calcein and, thus, monitoring of the green signal becomes an accurate surrogate for cell viability. After incubation with TP4, we found that cancer cells showed a greater loss of membrane integrity and reduction of cell viability compared to normal control cells (Figure 3A,B). Within a full dose–response testing, the loss of membrane integrity indicated by the EthD-1 signal occurred at comparable, albeit slightly lower concentrations of TP4 as indicated by the IC_50_ values (Figure 3C, Table 3). Of note, while TP4 reduced the active mitochondrial mass at a dose equal to the IC_50_ concentration, the reduction of the mitochondrial signal (Figure S3) was relatively less compared to the TP4-induced loss of cell viability. This result together with the equal potency of TP4 demonstrated in nonlipogenic and lipogenic pancreatic cancer cell lines suggest that mitochondrial targeting is not the major driving source of cell death by TP4.

**Table 3:** IC_*50*_ Values for Dual Calcein AM/EthD-1 Assay Dose Response after Incubation of PSN-1 Pancreatic Cancer Cells with TP4

PSN-1	Calcein	EthD-1
IC_50_ (μmol/L)	1.93 (ND)^a^	1.83 (ND)^a^

a ND: not determined.

**Figure 3:**
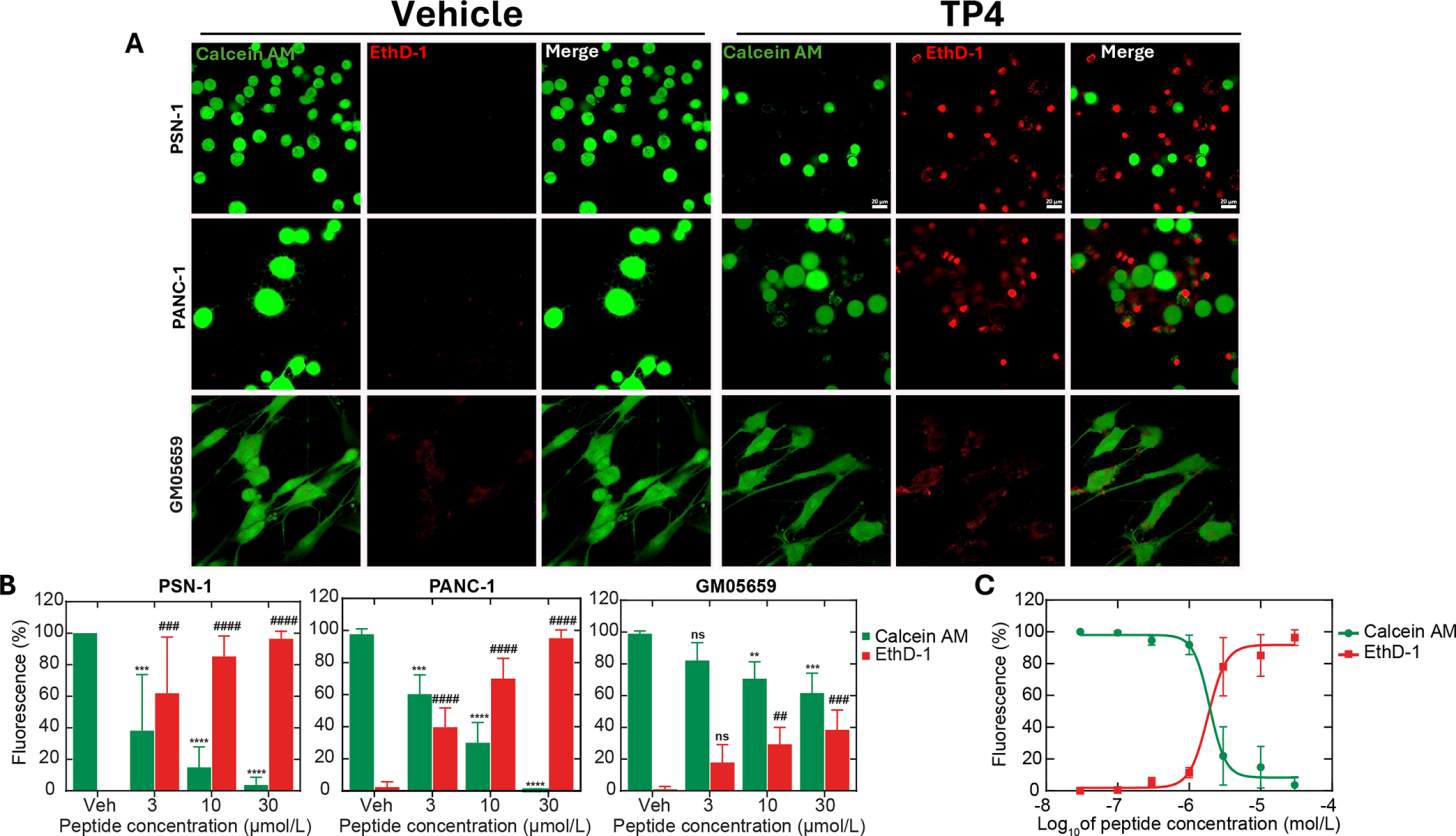
Effect of TP4 on membrane permeability and viability of pancreatic cancer cells (PANC-1 and PSN-1) and fibroblast (GM05659). (A) Representative confocal microscopy images of indicated cell lines treated with vehicle or 10 μmol/L of TP4 peptide for 24 h, followed by staining with membrane-impermeable Ethidium homodimer 1 (EthD-1; red) and calcein AM dye (green). Scale bar, 20 μm. (B) Quantification of calcein (green bars) and EthD-1-positive (red bars) cell fluorescence signal using ImageJ software for indicated TP4 concentrations; a minimum of 50 cells per triplicate were analyzed. Data were expressed as Mean ± SEM and analyzed by two-way ANOVA and multiple comparison test using GraphPad Prism software where ns for *p* > 0.05, Statistical ****, #### *p* < 0.0001, ***, ### *p* < 0.001, **, ## *p* < 0.05, * indicate comparisons of calcein, # of EhD-1 florescence signals. (C) Dose response curve calcein and EthD-1 in the presence of TP4.

### The Activity Profile of TP4 is Correlated with the Expression State of Regulators that Modulate Plasma Membrane Asymmetry

To provide additional insight into a potential contribution of compromised plasma membrane asymmetry in cancer cells and membrane disruption by TP4, we investigated correlations between peptide efficacy (i.e., IC_50_) with the expression status of flippases, floppases, and scramblases across the six tested cancer cell lines and found a strong correlation as shown in Figures 4 and S5. These enzymes maintain membrane asymmetry by regulating the distribution of anionic PS between the inner and outer leaflets. Flippases (type IV P-type ATPases) translocate PS from the outer to the inner leaflet while floppases mediate the reverse transport. Scramblases are energy-independent proteins that move lipids in both directions, which can lead to a rapid collapse of membrane asymmetry.^57^ Both enzyme families, which are frequently dysregulated in cancer, correlate with clinical outcomes, and have been proposed as biomarkers.^57–59^

**Figure 4:**
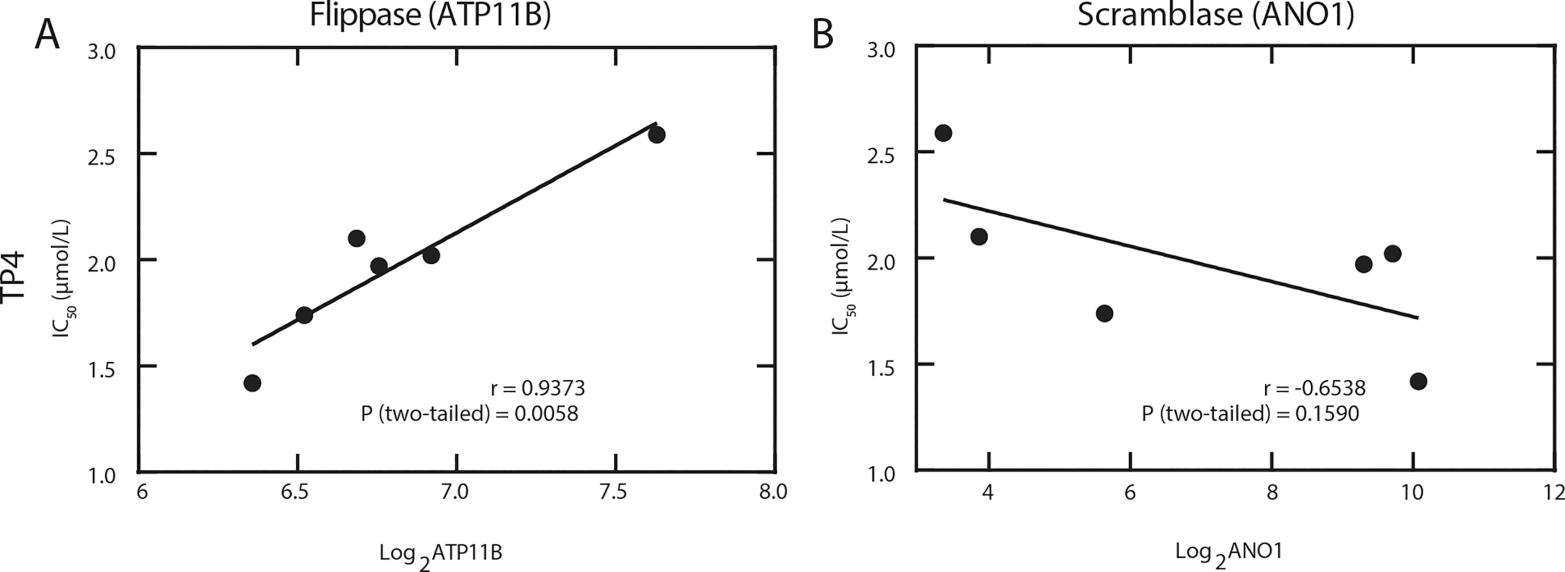
Expression levels of transmembrane proteins that control movement of phospholipids as a function of the activity of TP4. Gene expression levels indicate HumanHT-12 v4 expression BeadChip array signals, which were normalized to expression levels of housekeeping genes and log2-transformed. Correlation coefficient (*r*) and Pearson correlation coefficient test values are indicated. Data for all piscidins used for this assessment, with the corresponding flippase and scramblase levels, are provided in Figure S5. The gene expression levels for all enzymes can be found in Table S1.

Among the dysregulated flippases reported in pancreatic cancer (ATP11A, ATP11B, ATP8B1, ATP8A1, and ATP8A2),^57–59^ the expression status of ATP11B correlated most with the IC_50_ profile of the piscidin peptides (Figure 4, Figure S5), with TP4 showing a near-perfect linear positive correlation (*r* = 0.9373; *p* = 0.0058). Conversely, among the expression statuses of transmembrane proteins that function as scramblases (ANO1 and ANO6), ANO1 expression showed a negative correlation with the cell-based IC_50_ values for TP4. While these results support the notion that the externalization of negatively charged lipids or maintenance of plasma membrane asymmetry is associated with increased peptide activity (i.e lower IC_50_), the above findings represent associative rather than causative effects between gene expression levels and TP4 response.

In summary, the activity profile of TP4 in cell-based assays supports a reasonable therapeutic window. Furthermore, the peptide induces increased plasma membrane permeability in cancer cells compared to normal cells and its activity profile is associated with the expression state of regulators that flip PS to the outer membrane leaflet and control plasma membrane asymmetry. Informed by the biological data indicating that preferentially kills cancer cells via a membrane-mediated mechanism, we conducted a series of in-depth biophysical studies of TP4 to characterize, at the molecular level, both its mechanism of membrane disruption and cancer cell selectivity.

### Cholesterol and Negatively Charged Lipids Impact TP4 Binding to Model Membranes

To evaluate how different model membranes influence the secondary structure and membrane binding affinity of TP4, we performed CD spectroscopy with SUV titrations. To determine how negatively charged lipids influence peptide binding, membranes without and with POPS were assessed. We also wanted to understand how membrane fluidity, and acyl chain order impacts binding by using membranes with or without cholesterol. Thus, the membranes tested included POPC, a zwitterionic lipid; POPS, a negatively charged lipid; and/or cholesterol, all of which are present in cancer cell membranes.^14^ For each lipid composition, CD spectra were recorded at various P/L molar ratios ranging from 1:5 to 1:100 (Figure 5A). The piscidin family of peptides is known to form an α-helix in the presence of vesicles,^31,55,60^ characterized by the helix double minima at 208 and 222 nm.^61^ Therefore, we monitored the ellipticity at 222 nm across all P/L, and calculated the normalized mean residue ellipticity nMRE (Figure 5B). Normalization was done with respect to the ellipticity at 222 nm for peptide in buffer on the day of the measurement. An increasingly negative minima at 222 nm indicates an increase in α-helical content due to increasing amounts of peptides being bound to the lipid SUVs, while saturation correlates with the membrane surface having attracted the maximum amount of peptide. From this data, the dissociation constants (*K*_D_) and the number of lipids bound per TP4 molecule (*n*) were derived as described in the Methods section. The percent helicity was calculated as previously described,^62^ and all values are summarized in Table 4. Notably, these bilayer systems approximate cellular membrane compositions, and thus may not capture the dynamic complexity of living membranes. The model membranes used here are compositionally symmetric between leaflets, unlike biological membranes, which exhibit marked lipid asymmetry. A simple solution to produce mimics of the outer leaflet of healthy and cancer cell membranes was to measure separate bilayer samples: POPC and POPC/cholesterol representing healthy cell, and POPC/POPS and POPC/POPS/cholesterol for cancer cells. To these, TP4 was added in aqueous buffer and allowed to interact freely with the bilayers to produce the observed effects.

**Figure 5:**
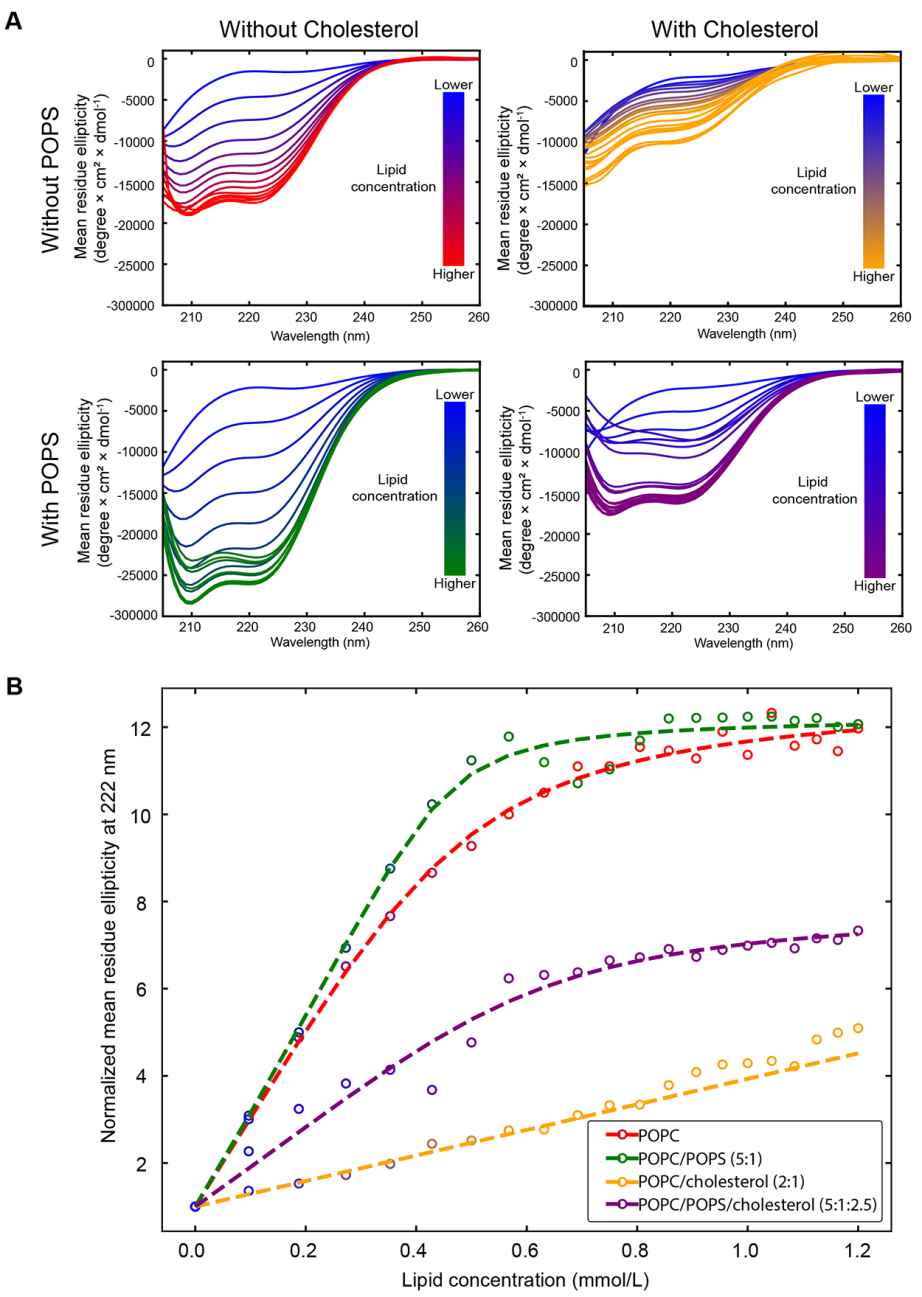
Circular dichroism data of TP4 upon titration of lipid vesicles. POPC-based lipid vesicles with or without POPS and/or cholesterol, were titrated into a solution of TP4. (A) CD spectra of TP4 were recorded following incremental additions of lipid vesicles to assess secondary structure changes, with CD spectra smoothened using a Gaussian fit. (B) Binding curves derived from the CD signal at 222 nm, with molar ellipticity values plotted against lipid concentration. Data were normalized to the signal at zero lipid on the day of the measurement and representative results from one independent experiment (*n* = 1) are shown. All fitting parameters are provided in Table 4.

**Table 4:** Fitting Parameters and Helical Content for TP4 in Lipid Vesicles^a^

Lipid	*K*_D_ (μmol/L)^b^	*n* ^c^	nMRE_max_	Helicity in buffer (%)^d^	Helicity in lipids (%)^d^
POPC	11.2 ± 9.6	19 ± 2	14.7	0.0	52.1
POPC/POPS (5:1)^e^	1.9 ± 1.2	25 ± 1	13.0	2.0	76.6
POPC/Chol (2:1)^e^	ND^f^	ND^f^	15.9	1.5	26.9
POPC/POPS/Chol (5:1:2.5)^e^	7.5 ± 5.6	23 ± 5	8.4	2.5	48.3

a Fitted parameters are reported with 68% confidence intervals.

b *K*_D_ indicates dissociation constant.

c *n* indicates the number of lipids bound to a peptide.

d Percent helicity is determined as previously defined by Ladokhin et al.^62^

e Ratios are reported as molar ratios.

f ND: not determined.

TP4 interacted with each of the model membranes examined, as evidenced by an increase in α-helical content with each addition of SUVs into the solution, but displayed differing binding characteristics. As seen in Figure 5, upon titration of a TP4 peptide solution with POPC or POPC/POPS SUVs, the peptide rapidly binds to the membranes, but showed a higher binding affinity for POPC/POPS (5:1) vesicles a composition relevant for the outer leaflet of cancer cell membranes.^14,63^ The 16% PS used in these experiments reflects the typical PS content of mammalian cells (10–20%).^12–15^ In healthy cells, PS is mostly restricted to the inner leaflet of healthy cell membranes, whereas in cancer cells it is exposed on the outer leaflet.^12,16–18^ The stronger binding of TP4 to the PS-containing membrane was corroborated by a dissociation constant (*K*_D_) almost 6 times lower than that of the pure POPC system, accompanied by a higher increase in α-helical content. These findings suggest that although cationic TP4 can associate with zwitterionic lipids, the presence of small amount of anionic lipids greatly increases recruitment of the peptide to the bilayer through electrostatic attraction.

In contrast, cholesterol markedly impairs the ability of TP4 to bind to membranes. Cholesterol is an essential component of both healthy and cancer cell membranes, contributing 20–40% of the total plasma membrane composition.^13,63–65^ Cholesterol is known to increase lipid packing and bending rigidity of bilayers, properties that are associated with limited binding and insertion of anticancer agents and AMPs.^66–68^ Consistent with those observations, TP4 exhibited both a modest binding and a reduced helical content in bilayers containing cholesterol compared to cholesterol-free bilayers (Figure 5). Note that POPC/cholesterol system does not appear to reach saturation in the range of P/L tested, thus fitting this binding curve with the proposed model (see Methods) results in unreliable values for *n* and *K*_D_ and, therefore, they were omitted. Of note, the inclusion of anionic POPS in the POPC/cholesterol bilayer restored some of the binding ability of TP4, but the binding remained significantly weaker than for POPC/POPS. Collectively, these findings highlight the inhibitory role of cholesterol on TP4-membrane interactions and emphasize the importance of negatively charged lipids (POPS) in promoting TP4 binding and folding, key features that may drive its selectivity and the strength of its action on cancer cell membranes.

### TP4 Dramatically Disrupts Membranes Containing Negatively Charged Lipids

To evaluate the effects of TP4 on bilayer structures, the peptide was incorporated into oriented lipid bilayer stacks (lamellar samples) of the same four lipid compositions as above and investigated with XRD. Lamellar samples were prepared at a P/L ratio of 1:25, as informed by the number of lipids per peptide determined from CD measurements and prior physical studies.^55^ This ratio is of biological and biophysical significance, as it provides a sufficient density of the peptide for a complete membrane coverage and a measurable manifestation of the membrane-perturbing activity through detection of permeabilization events, as demonstrated for other piscidins.^38,53,55^ Diffraction data were collected across incidence angles ranging from 0.4 to 6.0°, as Bragg reflections from the stacks of bilayers were not detected beyond this range (Figure 6). This is primarily due to the high thermal disorder of fluid bilayer systems.

**Figure 6:**
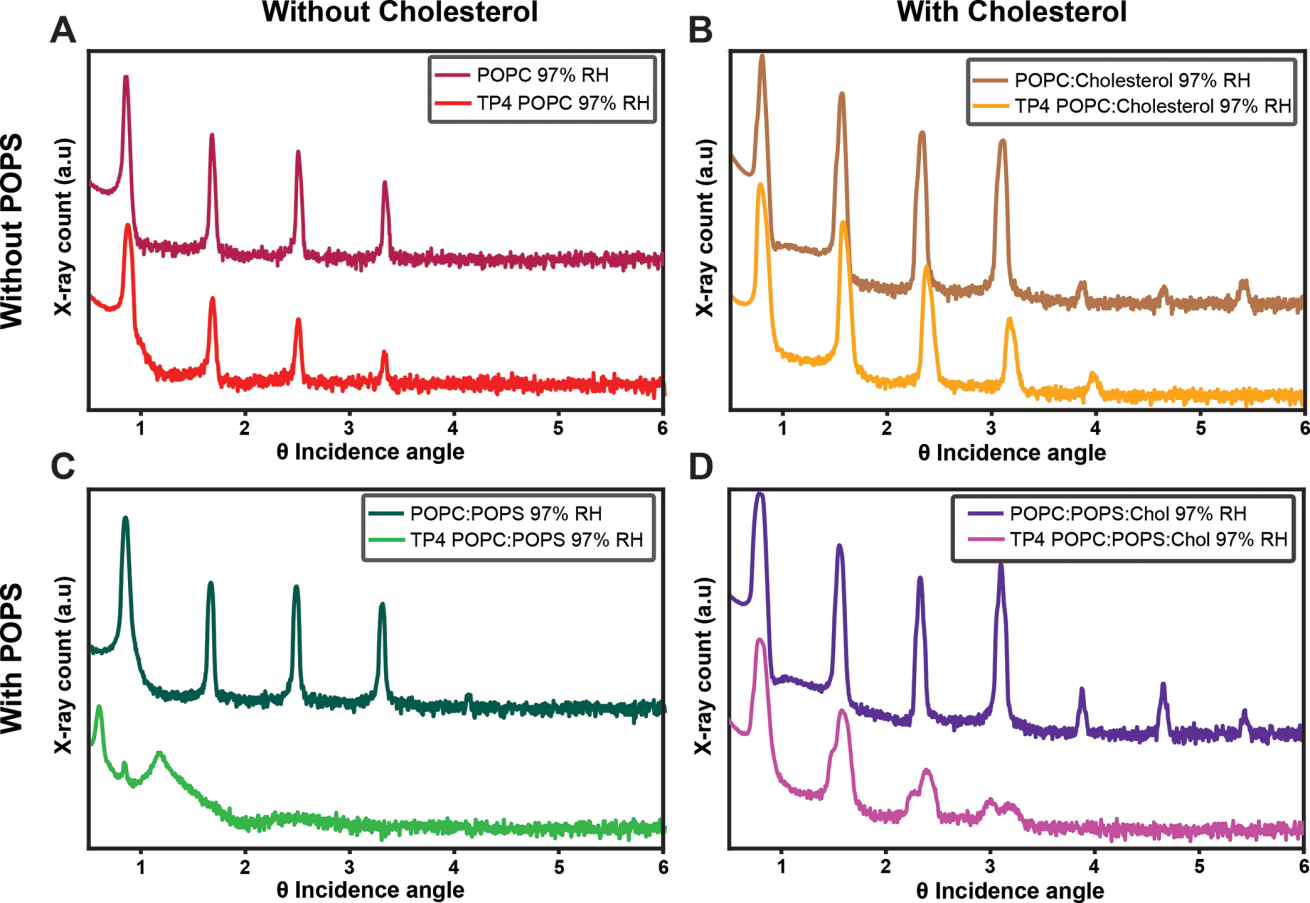
X-ray diffraction data of TP4 with lipid multilayers. Bragg diffraction from lamellar samples with and without TP4 for (A) POPC, (B) POPC/cholesterol (2:1), (C) POPC/POPS (5:1), and (D) POPC/POPS/cholesterol (5:1:2.5). All samples were produced at a P/L of 1:25 and were hydrated and measured at 97% relative humidity and 25 °C. Data for other hydration levels tested can be found in Figure S6 and Table S7.

XRD revealed perturbative effects caused by TP4 in the hydrated lamellar samples, but these effects varied between the different lipid compositions (Figure 6). In the absence of TP4, sets of equidistant Bragg peaks were observed for all lipid mixtures tested here. These peaks shift to lower angles (larger repeat distances) with increasing relative humidity, indicative of increased water uptake by the hygroscopic lipid headgroups (Figure S6). In the presence of TP4, apart from a reduction in peak intensities and a slight shift in peak positions relative to the pure bilayers, the Bragg diffraction signal is fairly well preserved for POPC (Figure 6A) and POPC/cholesterol (Figure 6B), indicating superficial perturbations to the bilayer structure. However, the lack of higher-order reflections suggests that TP4 introduces lattice defects and disrupts lamellar order.

In contrast, the inclusion of the negatively charged POPS lipid resulted in significant reorganization and bilayer perturbations in the presence of TP4. This was observed as peak splitting and broadening that culminated with an almost complete loss of Bragg peaks for POPC/POPS membranes (Figure 6C). In POPC/POPS/cholesterol membranes, two sets of peaks emerge in the presence of TP4 (Figure 6D), indicating that (at least) two repeat distances are present, with values of ∼58.6 and ∼55.3 Å (Table S8). This can be explained by a separation into domains under the action of TP4: a POPS/TP4-rich domains coexisting with a POPC/cholesterol-rich (PS-depleted) region. The separation is expected to be caused by TP4 binding strongly to PS but weakly to cholesterol-containing bilayers, as shown by the CD measurements (Figure 5). The separated domains have very different properties (bilayer thicknesses and hydration capacities) and can create columnar stacks of like bilayers. The lines of tension created at the boundaries between these domains can cause severe membrane disruptions and facilitate the insertion of TP4.^38,69,70^ For instance, the larger repeat spacing of 58.6 Å for POPC/POPS/cholesterol with TP4, splitting away from the original set of peaks (Figure 6D), is most likely due to POPS/TP4-rich domains that can absorb a high amount of water, leading to significant swelling and hence, larger repeat distance. The water retention in bilayer systems with TP4 is analyzed in more detail below, using ND data.

### TP4 is Embedded into the Hydrocarbon Layer of a Fluid Bilayer

To determine the location of the TP4 peptide in membranes containing anionic lipids, NR experiments were performed. Solid-supported lipid bilayers composed of DOPC/DOPS (3:1 molar ratio) were adsorbed onto a silicon substrate, and the neutron reflectivity at the interface was measured before and after exposure to 3 × 10^–6^ mol/L TP4 in 10 mM Tris at pH 7. The neutron reflectivity curves (Figure S7) were fit using a composition space model of the bilayer that accounts for the stoichiometry, connectivity, and known molecular volumes and formulas of the lipid headgroups and acyl chains, while allowing the peptide to insert in and around the bilayer without any structural assumptions (see Methods). The resulting volume occupancy profiles of the bilayer before (Figure 7A) and after (Figure 7B) exposure to the peptide reveal the peptide location upon insertion and equilibration into the membrane.

**Figure 7:**
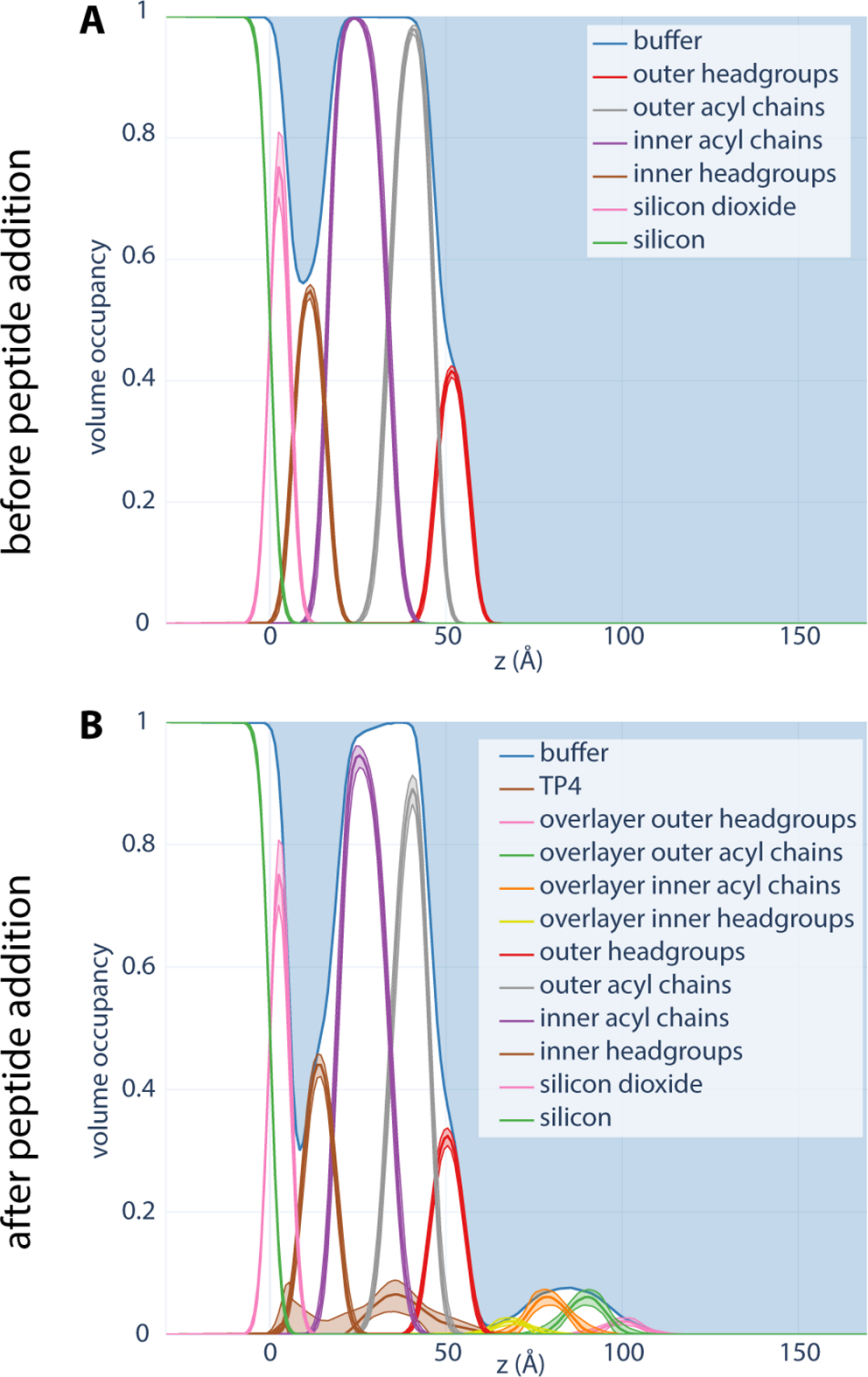
Volume occupancy distributions representing the structure of the bilayer as observed by neutron reflectometry. DOPC/DOPS (3:1) vesicles were allowed to adsorb onto a silicon wafer until fully covered. TP4 was injected using a syringe pump. Free lipids and TP4 were washed off the wafer between each step. Results were collected (A) before and (B) after adding TP4.

NR measurements highlight several key features of TP4’s interaction with DOPC/DOPS (3:1) membranes. Prior to adding TP4, the bilayer is characterized to have an initial thickness (average distance from the center of the bilayer to the headgroups) of 30.44_–0.08_^+0.07^ Å. After the addition of TP4, the bilayer thins by 3.68_–0.24_^+0.26^ Å (12% reduction), indicating peptide-induced bilayer thinning (Table S8). Additionally, these measurements allowed us to observe the fraction occupancy of water, substrate, peptide, and various membrane components forming the bilayer. After the addition of TP4, we observed a greater water occupancy between the substrate and the inner headgroup, compared to the control without TP4. A separate experiment using TP4 without the polyarginine tail (Figure S9) showed comparable thinning and membrane insertion to TP4, but only a modest increase in water occupancy near the substrate, suggesting that the polyarginine may play a role in transporting water into the membrane. Furthermore, NR profiles indicate the appearance of a second layer above the bilayer after TP4 addition, which is absent in the the the profile of the polyarginine-truncated TP4 variant. The formation of a second layer, coupled with the decreased volume occupancy for all membrane components after adding TP4, suggests that the polyarginine tail may anchor displaced lipids above the supported bilayer’s surface.

### TP4 Recruits Water and Diffuses the Bilayer-Water Interface

To characterize and quantify the structural perturbations induced by TP4 upon membrane binding, we employed ND. In this case, POPC was used as the model membrane since TP4 associates favorably with POPC and the diffraction signal from a mixture of POPC/POPS with TP4 would not be feasible for analysis, as seen with XRD (Figure 6). Lamellar samples of POPC and TP4/POPC (1:25) were investigated for several samples hydrated from the vapor phase, using deuterium labeling in the water of hydration (i.e.,^2^H_2_O vapor) and lipid acyl chain. The resulting scattering length density (SLD) profiles and their amplitudes (Figure 8), calibrated using deuterium labeling, reveal true spatial redistributions of density within the bilayer and enable deconvolution of the relative contributions from peptide, lipid, and water components.

**Figure 8:**
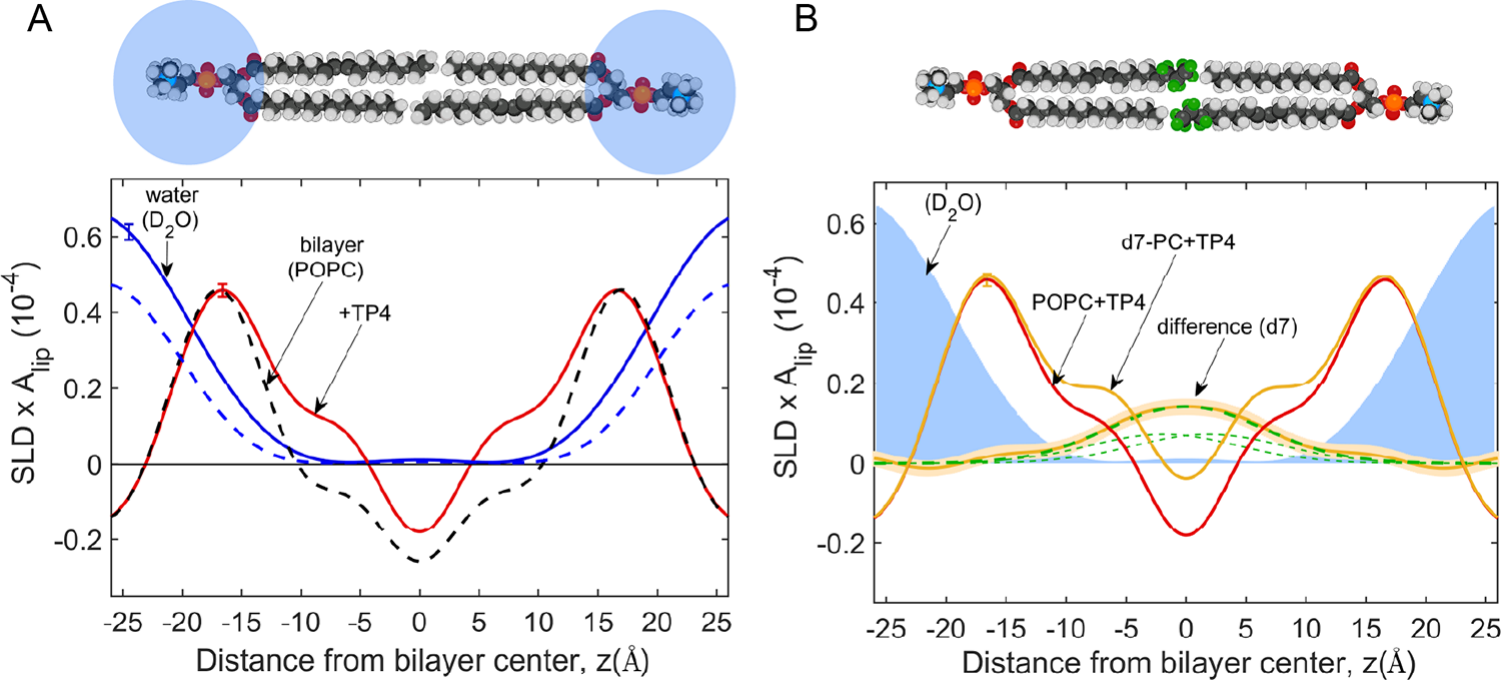
Neutron diffraction density profiles of bilayers with and without TP4. (A) Neutron scattering length density (SLD) profiles of a neat POPC bilayer (black, dashed) with its water distribution (blue, dashed), and a POPC/TP4 bilayer (red) with its corresponding water distribution (blue, solid). The profiles corresponding to only one bilayer are shown out of the stack of bilayers. Error bars illustrate the magnitude of the uncertainties (±1 standard deviation) (see 2 Methods). (B) Bilayer profiles of POPC with TP4 (red) and a mixture of POPC and tail deuterated PC (D7-PC), 1:5 POPC/D7-PC (yellow). The difference between the data from deuterated and protonated equivalent samples (deuterium difference) shows the distribution of deuterium in the bilayer. The water profile (shaded blue areas) for POPC/TP4 bilayer complex was determined by deuterium difference using H_2_O/^2^H_2_O contrast variation. The P/L was 1:25 in all samples. The D7 profile and its uncertainty (yellow band) could be best fit by a pair of Gaussian distributions (green dotted lines) positioned symmetrically at −1.87 (±0.3) Å and +1.87 (±0.3) Å from the bilayer center, and a FWHM = 15 (±0.3) Å. All uncertainties correspond to 1 SD. Lipid structures representations were made with BioRender.

The first observation is that TP4 incorporation into POPC bilayers induces a pronounced increase in scattering length density within the interior of the bilayer, particularly in the region from *z* = 0–15 Å, relative to the bilayer center. Because peptides have higher neutron scattering lengths than the lipid hydrocarbon chains, but comparable to lipid phosphate headgroups, the increase in hydrocarbon SLD in the presence of TP4 (Figure 8A) can derive from two types of contributions: direct insertion of TP4 into the hydrophobic core and/or displacement or pulling of POPC headgroups deeper into the bilayer via hydrogen bonding with TP4, leading to bilayer surface deformations. These deformations are typically associated with a decrease in bilayer thickness, as previously demonstrated for P1 and P3,^53^ and are evident here as a slight shift of the phosphate group peaks toward the bilayer interior for bilayers with TP4, compared to a neat POPC bilayer profile. However, there is an overall increase in the lamellar repeat spacing (bilayer thickness + water layer) from 53.2 Å in neat POPC to 54.9 Å with TP4. This increase can be attributed to pronounced water accretion in bilayers with TP4. Collectively, these changes are consistent with our volume occupancy results derived from NR, which show that TP4 resides predominantly in the acyl chain region of the bilayer. However, the ND profiles more clearly reveal the bilayer structure and water redistributions under the action of TP4.

To further investigate the role of the polyarginine motif at the C-terminal end of TP4, a segment that is absent from P1 or P3, we examined how TP4 impacts membrane hydration. The guanidium side chain of arginine is known to form strong hydrogen bonds with phosphates and water molecules^71,72^ thus, we want to examine the hydration properties of TP4 in the context of the bilayer. Due to the hygroscopic nature of phospholipid headgroups, under controlled-humidity environments, lipid multilayers absorb water that accumulates around the headgroups and in between adjacent bilayers. However, the presence of a peptide can dramatically change the distribution of water in the system, as seen in Figure 8A. Taking advantage of the deuterium contrasts, we quantified the amount of water accumulated in bilayers with TP4 at a P/L = 1:25. Based on the CD measurements, one TP4 peptide binds to approximately 19 POPC lipids and thus, at a P/L = 1:25, we can assume that all peptides are bound. Under that assumption, we incorporated D7-PC (a tail-deuterated PC lipid) into lamellar samples of known composition as a standard for calibration of the water peaks, found by H_2_O/^2^H_2_O exchange (see Methods and Figure S9). The number of waters per POPC headgroup has been determined before to be 9.4 waters/lipid^73,74^ at 93% RH and 23 °C. Here, under the same conditions, we determined that 114 ± 15 water molecules are bound per peptide, in addition to the water bound to the PC headgroups. This takes into account the ∼57 exchangeable protons per TP4,^75^ which contribute to the measured signal during isotope exchange. Notably, TP4 retains a high hydration level under partial hydration conditions and carries water into the membrane as it inserts. The resulting water profile extends well into the hydrocarbon region, overlapping with the broad distribution of the deuterated acyl chain termini (D7-PC). The results support a model in which TP4 binding causes bilayer deformations and blending of the water with the hydrocarbon chains, thus reducing the hydrophobic gap and facilitating the membrane permeabilization and the translocation of the cationic peptide across the membrane.

### Molecular Dynamics Simulations Reveal Possible Conformations of TP4 in a Fluid Bilayer

To visualize the molecular connections that TP4 may establish with the bilayer surfaces and the hydrocarbon, MD simulations were performed. Simulations were built using the CHARM-GUI membrane builder for a POPC/POPS (5:1) system and a P/L ratio of 1:100, reproducing the composition used in CD, for which a helical content of 77% was found. At these surface densities, the peptides are expected to insert into the bilayer in the folded conformation and reside on the bilayer surface. The results from the final all-atom simulations are shown in Figure 8.

Snapshots of the equilibrated system of the enhanced MD simulations reveal several key features of TP4-membrane interactions. The images of the TP4-POPC/POPS ensemble (Figure 9A), together with the electron density profile (Figure 9B), show that three out of the four peptides are fully inserted into the bilayer, with hydrophobic residues pointing toward the center of the bilayer, while one of the peptides is kinetically trapped into a state not entirely buried in the bilayer. These observations align with experimental NR results, supporting that TP4 is mostly buried in the hydrocarbon region of the membrane.

**Figure 9:**
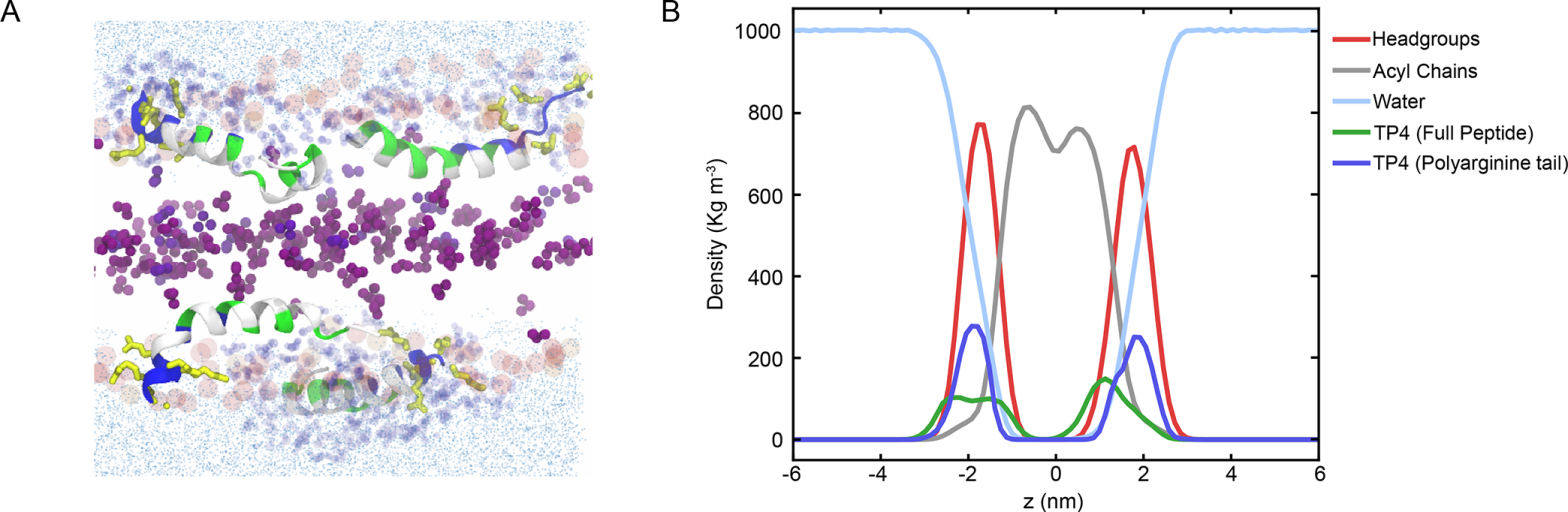
Molecular dynamics simulation results for the TP4-membrane system. Simulations were run for 500 ns using a HMMM-based model generated with the CHARMM-GUI membrane builder, followed by 100 ns of all-atom simulations. (A) Rendering of the peptide-bilayer system at the end of the 500 ns simulation run. The peptide backbone is colored blue (cationic side chains), white (hydrophobic side chains), and green (all other side chains); the side chains for the polyarginine tail are shown as yellow stick representations. Lipid headgroups are represented as spheres (POPC in red, POPS in orange), terminal methyl groups of the lipid tails are depicted as purple spheres, and the rest of the acyl chains were not shown to improve visualization of the peptides. Water molecules interacting closely with the peptide are shown as dark blue spheres and bulk water molecules as light blue spheres. (B) Electron density profiles of the various groups located along the lipid bilayer. Data showing the electron density profile for the lipid bilayer without the peptide are shown in Figure S10.

Additionally, to understand the role of the acyl chains in TP4 insertion into the membrane, we calculated the distribution of the terminal methyl hydrogen of the oleoyl 18:1 chains to match the ND profile for the D7-PC group. Acyl chains in the bilayer core are highly disordered,^76^ and their flexibility allows terminal methyl groups to sample positions that extend toward the bilayer surface.^41^ Consistent with this, MD simulations (Figure 9A) reveal that while most terminal methyls are confined to the center of the bilayer, some of them can display motions that bring them close to the bilayer-water interface and the peptide. This observation is in agreement with ND profiles for the deuterated acyl terminus (D7-PC) (Figure 8A), indicating that the high degree of disorder, partly created by the presence of the peptide, can blur the hydrophobic–hydrophilic segregation of the bilayer interfaces. This is exacerbated by the peptide drawing water with it as it distributes within those interfaces, being partly buried in the hydrocarbon region. Overall, the results suggest that van der Waals interactions between the highly hydrophobic acyl chain termini and the hydrophobic surface of the amphipathic peptide facilitate anchoring of the peptide at the hydrophobic–hydrophilic interface of the bilayer.

Finally, to directly compare with ND experimental results, we analyzed the hydration properties of TP4 in the context of the bilayer using radial distribution function analysis to identify water closely bound to TP4 peptide and, in particular, the polyarginine tail (Table 5). We calculated that two hydration shells around the peptide contain 106 ± 26.2 water molecules, which closely matches the 114 ± 15 molecules of bound water as calculated by ND. Altogether, the MD simulation results support experimentally observed phenomena and provide a molecular-level visualization for the interactions of TP4 with membranes.

**Table 5:** Radial Distribution Function Analysis of Water Molecules Near TP4 and the Polyarginine Tail^a^

TP4 (Polyarginine tail)			TP4 (Full-length peptide)	
Shell No	radius for shell (Å)	Number of water molecules	radius for shell (Å)	Number of water molecules
First Shell	3.10 ± 0.03	9.01 ± 2.30	3.05 ± 0.02	31.6 ± 4.51
Second Shell	4.40 ± 0.06	31.3 ± 5.07	4.32 ± 0.06	106 ± 26.2
Third Shell	4.63 ± 0.29	35.4 ± 6.60	5.47 ± 0.08	155 ± 41.2

a Hydration shell radii and the number of water molecules within each shell for both TP4 tail as well as the whole TP4 peptide.

## Discussion

In this study, we used biological and biophysical methods to demonstrate the importance of anionic lipids for the selective anticancer activity of TP4 and elucidate the molecular determinants underpinning its promising potency. TP4 was selected due to its demonstrated cytotoxicity against a broad spectrum of cancers, including triple-negative breast,^30,33^ bladder,^34^ lung cancer,^28^ and the pancreas cancer featured in this study. While AMPs such as TP4 have reached in vivo testing in prostate and breast cancer models,^30,77^ the narrow therapeutic window, which depends on biophysical differences between normal and malignant membranes, is a major hurdle toward further preclinical development of membrane-targeting peptides.^78^ To understand at the molecular level how TP4 specifically interact with cancer cells, a characteristic that might be leveraged for improved selection of cancer types for this emerging therapeutic approach, we performed a series of advanced biophysical and molecular interrogations on PC-based model membranes, PC being the major lipid of mammalian cell plasma membranes.^79^ To these model bilayers, cholesterol and PS lipids were added in proportions found in the plasma membranes of healthy and cancer cells. Next, we discuss the biological features that promote the interaction of AMPs such as TP4 with cancer cell membranes as well as the molecular determinants and distinctive behaviors of TP4 that are associated with its selective anticancer activity.

### Biological Features that Facilitate the Interactions of AMPs Such as TP4 with Cancer Cell Membranes

#### Negatively Charged Lipids as Selectivity Factors for Cancer Cells

One distinguishing feature of healthy mammalian cells is the asymmetric distribution of lipids across the plasma membrane, with zwitterionic lipids such as POPC enriched in the outer leaflet and negatively charged lipids like POPS localized to the inner leaflet,^12,80^ where they can reach up to 20% of the total phospholipid, based on lipidomic and NMR studies.^12,14,79,81^ Interestingly, cancer cells often display disrupted lipid asymmetry, characterized by the externalization of anionic phospholipids, such as POPS, to the outer leaflet. This shift toward a negatively charged surface can facilitate immune evasion by suppressing an anti-inflammatory response, mimicking apoptotic cell signatures.^18^ Ting et al. previously demonstrated that TP4 exhibits preferential cytotoxicity toward triple-negative breast cancer cells compared to human fibroblasts.^30^ In the present study, we extend these findings by confirming the selectivity of TP4 across diverse pancreatic cancer cell lines compared to normal pancreatic epithelial cells and primary human fibroblasts (Figure 4). During apoptosis, scramblase proteins such as the Xk-related family of proteins are activated, reducing asymmetry by externalizing POPS lipids and signaling phagocytes to remove these cells.^16,17^ In contrast, flippase proteins, which are primarily responsible for modulating membrane function, maintain electrostatic asymmetry by transporting negatively charged phospholipids from the outer leaflet to the inner leaflet.^57^ In the present study, we show that increased expression of the flippase, ATP11B, which reduces the negative charge on the outer-leaflet, correlates with decreased TP4 activity, whereas reduced expression of the scramblase ANO1 that mediates the reverse process correlates with increased TP4 activity (Figure 4). These relationships, in large also observed for P1 and P1R5, reflect correlations between IC_50_ values and enzyme expression across distinct pancreatic cancer cell lines and do not imply direct causation. The impact of manipulation of ATP11B and ANO1 levels via siRNA knockdown on TP4 response profiles could not be successfully evaluated due to target knockdown-associated toxicity (data not shown); across different cancer models, ATP11B and ANO1 have been shown to control cell proliferation and cell viability,^82–86^ and the induced cell death upon silencing of these two regulators invalidated the impact of adding TP4. Nonetheless, a possible role of membrane asymmetry-regulating enzymes on the response of TP4 is supported by our CD and XRD measurements using POPC-based model membranes in which ∼33% cholesterol and ∼16% POPS (molar fraction in total lipid) were sequentially incorporated to mimic the lipid composition of the outer leaflet of healthy versus cancerous plasma membranes. The data show that while TP4 binds all tested membranes, cholesterol impedes the binding and insertion of TP4 into the bilayer (Figure 5). In contrast, incorporating 16% POPS overcomes this protective effect, whether or not cholesterol is present, allowing for TP4-induced membrane disruption (Figure 7), likely due to electrostatic attraction between the positively charged peptide and negatively charged membranes. Interestingly, similar to TP4, the wasp peptide polybia-MP1 (IDWKKLLDAAKQIL), which exerts anticancer activity albeit at significantly lower efficacy (IC_50_ > 15 μmol/L),^87^ also exhibits preferential binding to membrane containing anionic PS.^88,89^ Together, the biological and biophysical data support the conclusion that exposure of POPS on the outer leaflet makes the membrane more susceptible to structural perturbation by AMPs such as TP 4, leading to the relatively higher cytotoxic effect on cancer versus normal cells.

Notably, ATP11B has recently been identified as a bona fide drug target in pancreatic cancer, with the LTX-315 peptide potentially inhibiting ATP11B, resulting in improved prognosis.^90^ The expression status of flippase, scramblase, and possible floppase enzymes, which are frequently dysregulated in solid organ cancers, could potentially become a biomarker for peptide treatment during future preclinical development.

While this study focuses on POPS, other negatively charged lipids (phosphatidic acid, phosphatidylinositol, and phosphatidylglycerol) and membrane proteins, many of which are heavily glycosylated with terminal sialic acids, contribute to the net negative charge on the surface of mammalian cells and are bound to facilitate the interactions of TP4 with cancer cell membranes.^14,91^ We have previously established that TP4 interacts favorably with negatively charged lipids such as POPG and LPS.^31^ Through biophysical assays, we reveal that while TP4 binds to all tested POPC-based membranes, significant structural disruption occurs only in the presence of POPS-containing membranes. These results suggest that, while cancer cells may exploit negatively charged exterior membranes for immune evasion, this same feature enables TP4 to selectively target and disrupt them, highlighting its future potential as a selective, membrane-active anticancer therapeutic.

#### Mitochondrial Disruption may be a Secondary Effect of the Anticancer Activity Exerted by TP4

Determining the primary anticancer effect of AMPs has been challenging. Intracellular targets may only be accessible after membrane lysis, rather than through direct peptide translocation across membranes, making it challenging to distinguish between the primary drivers of cytotoxicity and secondary downstream effects. To examine whether a primary antimitochondrial mechanism is driving the anticancer cell activity of piscidin peptides, we compared the activity of piscidins against lipogenic and nonlipogenic cells. Lipogenic cell lines have an increased mitochondrial mass, rely on fatty acid synthesis and oxidative phosphorylation, and are more sensitive to inhibitors of mitochondrial function such as fatty acid synthase (FASN) or stearoyl CoA desaturase 1 (SCD1) inhibitors.^54^ If piscidins exerted their mechanism of action primarily by targeting mitochondria, we would expect heightened sensitivity in lipogenic cancer cell lines. However, from all the piscidins tested, we did not observe significant differences in activity against lipogenic and nonlipogenic cancer cells. In addition, when we used TP4 at concentrations sufficient to achieve loss of cell viability (Figures 1, 2) and loss of membrane integrity (Figure 3), the reduction of active mitochondrial mass (as detected using a fluorescent readout) was not commensurate. While these findings do not exclude antimitochondrial mechanisms independent of mitochondrial mass and metabolism, they support that the previously observed mitochondrial dysfunctions associated with AMP administration^28,30,32–34^ might represent secondary effects due to a yet-to-be-defined primary mechanism. In future work, it would be insightful to examine necrotic versus apoptotic pathways and further strengthen the relationship between the anticancer activity of TP4 and its plasma membrane disruption.

### Membrane Properties and Distinct Peptide-Lipid Interactions Linked with Membrane Activity

#### Membrane Heterogeneity and Fluidity as Cofactors

The anticancer action of AMPs such as TP4 starts at the plasma membrane—the primary barrier that the peptides have to breach before reaching intracellular targets. Although far from capturing the complexity of natural cell membranes, model lipid bilayers based on PC, PS, and cholesterol are amenable to structural and biophysical investigations that would not be possible in natural cells. They are used here to identify key molecular features and parse out specific interactions that are relevant for mechanisms of action of TP4 and related MAPs against cancer cells.

The combined CD and XRD results show that TP4 binds to some extent to all membranes tested; however, cholesterol-containing membranes exhibit reduced peptide-induced membrane perturbation and binding compared to cholesterol-free membranes. Similar observations were made in other studies of AMPs.^66–68^ Cholesterol, a key component of mammalian membranes, is known to decrease bilayer elasticity and increase bilayer cohesiveness.^68^ It accounts for a significant portion (20–40%) of the total lipid in plasma membranes,^13,14^ though the interleaflet distribution of cholesterol is elusive,^92,93^ possibly due to its ability to exchange rapidly between the two leaflets to adjust to an everchanging lipid profile. While there are no specific lipid profiles that differentiate the cholesterol content between healthy and cancer cells,^93^ some evidence suggests that healthy cells maintain a higher fraction of cholesterol in the outer leaflet, where cholesterol associates with sphingomyelin and saturated lipids to form liquid-ordered domains (see ref 13 and references therein).

The possible effects of cholesterol on the lytic activity of MAPs have been discussed at length elsewhere, in the context of the archetypal MAP, melittin, for instance.^68,94^ Here, we propose an explanation for the hindered binding of TP4 at membranes with cholesterol, based on experimental evidence from our previous and current ND measurements, corroborated by MD simulations.^41^ In our previous studies,^41^ using specific deuteration of acyl-chain methyl groups and ND measurements, we showed that in the liquid-disordered (L_d_) state of a dioleoyl-phosphocholine (DOPC) bilayer, the chain disorder is so high that more than 20% of the methyl groups are in intimate contact with the lipid headgroups and water. The presence of the rigid cholesterol molecules in the L_d_ phase of a lipid bilayer impedes motions in the hydrocarbon acyl chains leading to the formation of liquid ordered (L_o_) states.^95–97^ Thus, the ordering of the acyl chains by cholesterol resulted in a measurable retraction of the methyl groups away from the bilayer-water interface, concomitant with a significant thickening of the hydrocarbon region.^41^ A similar degree of disorder is seen here with ND, at least for the oleoyl chains (Figure 8B), and can be better visualized with all-atom MD simulations (Figure 9A). A snapshot of the TP4 equilibrated in a POPC/POPS (5:1) bilayer highlighting the terminal methyls of POPC and POPS, shows that the chain termini explore the entire hydrocarbon region space up to the water interface. This implies that contacts form continuously between the hydrophobic surfaces of the amphipathic TP4 and the highly hydrophobic terminal methyls of the acyl chains. As a consequence, the chain termini can exert a strong pulling effect on the peptide, aiding its insertion into the hydrocarbon. As we have shown previously, cholesterol would oppose this effect by confining the terminal methyls to the bilayer center and thickening the hydrocarbon region.^41^ This provides a direct, structural, molecular basis for the reduced partitioning and activity of AMP in membranes with cholesterol. As such, a chain ordering effect hampers the creation of strong hydrophobic interactions that would allow anchoring of AMPs at the hydrocarbon–water interface.

Interestingly some peptides, including the piscidin homologues P1 and P3, that lack the polyarginine tail, can overcome the cholesterol barrier by inducing phase separation into cholesterol-rich and cholesterol-deficient domains, facilitating membrane disruption by selectively partitioning into L_d_ domains.^38,98^ Unlike P1 and P3, which rely on modulating membrane fluidity to induce phase separation even in zwitterionic-only membranes, TP4 appears to drive domain separation primarily through electrostatic interactions since the peptide requires an anionic lipid to produce this effect. However, this does not exclude other membrane segregation events, such as L_o_–L_d_ domain formation, which may occur at higher TP4 concentrations than tested here. Overall, the segregation into domains highlights the need for heterogeneity in membrane composition for some peptides to exhibit membrane activity.^99–101^

#### Depth Distribution and Hydration Profiles of TP4 that Lead to Membrane Permeabilization

NR performed on a planar, fluid bilayer of DOPC/DOPS (5:1) reveals that, when injected on one side of the bilayer, TP4 can insert into the acyl region of the bilayer and translocate through the bilayer to populate the other bilayer surface, bringing water with it (Figure 7). A significant fraction of the peptides is found buried in the hydrocarbon region. Notably, the injection of the peptide at 3 μmol/L is expected to yield, initially, a much higher concentration at the bilayer surface probably leading to a crowding effect, more closely resembling situations found in bacterial susceptibility tests. From the volume occupancies we estimated that, at equilibrium, about 2/3 of the injected TP4 distributed in the bilayer (at a P/L of 1/14), while the remaining 1/3 was, presumably, dispersed into the bulk solvent. However, under the conditions of the NR experiment it is difficult to fully quantify and appreciate the gradual changes to the bilayer structure and hydration profile brought by the peptide.

For a higher resolution than possible by NR and to observe structural changes under more controlled conditions, we used ND. When TP4 was incorporated in POPC oriented multilayers at a P/L of 1:25, we explored a situation corresponding to an almost complete bilayer coverage with peptides,^55^ since one peptide would binds to roughly 19 POPC molecules, based on CD (Table 3). To quantify the amount of water tightly associated with the peptide, the multilayers were hydrated from the vapor phase, under controlled humidity. At 93% relative humidity, we determined that TP4 carries along more than 100 water molecules into the membrane. This is corroborated by the MD simulations, which indicate that this amount of water corresponds to more than two hydration shells for the entire peptide, with the polyarginine tail retaining a large fraction of the water, and the NR results that show swelling of the water profile in the submembrane space for TP4 but not for TP4-noR5 (Figures 7 and S8; Table S9). Taken together, the profiles obtained from NR, ND and MD simulations offer a compelling image of the TP4/bilayer complex at the point of membrane breaching and beyond. Thus, in the more dilute regime, the TP4 peptides can be located in the hydrocarbon just under the lipid phosphate group line (MD simulations, Figure 9). These initial position and conformation develop into further insertion of TP4 and associated water into the hydrocarbon region (ND, Figure 8) and culminate with the peptide populating the entire hydrocarbon region and closing the hydrophobic gap (NR, Figure 7).

#### Distinct Membrane-Interactions Associated with the Polyarginine Motif

The membrane activity of TP4 starts with an efficient recruitment of the highly charged peptide at PS-containing membranes followed by membrane destabilization through lipid clustering, thinning of the hydrocarbon region, water retention and convoluted deformations. Interestingly, the membrane effects induced by TP4 are manifested most strongly in heterogeneous membranes. Due to its modular design, which features a helical portion followed by a flexible R5 tail, TP4 can severely stress the membrane through strong but opposing hydrophilic and hydrophobic interactions (e.g., the electrostatic attraction with the anionic lipid headgroups, which may act to retain the peptides on the membrane surface, oppose the hydrophobic pull of the peptide into the hydrocarbon). In prior studies,^31^ comparing data from TP4 and its truncated homologue (TP4-noR5), we established that the R5 segment is the main element responsible for the separation of anionic lipid domains in membranes, leading to membrane structural destabilization. These effects can manifest at either bacterial plasma membranes, which contain a high fraction of the anionic phosphatidylglycerol (PG) lipid, or cancer cell membranes that expose PS lipids on their outer leaflet. In the present study, using neutron measurements, we also reveal how the polyarginine tail promotes water recruitment in bilayers, as part of its multifaceted mechanisms for membrane disruption, a feature we did not previously observe with P1 or P3.^53^ While the polyarginine motif is not strictly required for anticancer efficacy, here, we present it as a primary driver of enhanced membrane disruption. For instance, although P1 and P3 exhibit moderate activity against various pancreatic cancer cell lines, their potency consistently falls short of TP4 (Figure 1, Table 1). LL-37 (LLGDFFRKSKEKIGKEFKRIVQRIKDFLRNLVPRTES), a well-characterized AMP, was previously shown to have an IC_50_ of 10.17 μmol/L against PANC-1 cancer cells,^102^ nearly 4× greater than what we report for TP4 (IC_50_ = 2.59 μmol/L). While both peptides exhibit membrane-disrupting activity near the bilayer interface,^103^ the reduced potency of LL-37 is likely attributed to either negatively charged aspartate and glutamate residues, which may attenuate membrane binding by reducing strong electrostatic interactions between highly cationic peptides and anionic membranes, and/or the absence of a polyarginine motif. This claim is supported by our observation that appending a polyarginine motif to the C-terminus of P1 (P1-R5) increases activity across all pancreatic cancer cell lines tested, identifying the polyarginine motif as the defining feature responsible for the superior performance of TP4 relative to other piscidin homologues.

Notably, polyarginine segments are present in several highly active MAPs (see ref 31 and references therein) and cell-penetrating peptides (CPPs).^104,105^ For example, melittin (GIGAVLKVLTTGLPALISWIKRKRQQ)—the principal peptide toxin in honeybee venom—possesses a polybasic motif (KRKR) near its C-terminus, similar to the TP4 polyarginine tail. As TP4, melittin inserts into the acyl chain region to execute its disruptive mechanism; however as a toxin, it lacks specificity.^106^ Importantly, this structural similarity correlates with their comparably strong activity against PANC-1 cells; melittin has an IC_50_ of 2.79 μmol/L,^107^ which is close to that for TP4 (IC_50_ = 2.59 μmol/L). For both AMPs and CPPs, the polyarginine segments play key roles in their binding to anionic lipids, membrane activity,^31^ and ability translocate into the cytoplasm.^105^ This has been attributed to the unique structural and chemical properties of the guanidinium side chain, which promotes strong hydrogen bonding with phospholipid headgroups and water molecules, while also enabling the shedding of water as needed to transfer through hydrophobic environments.^72,108^ These types of interactions promote the bilayer deformations and local membrane curvatures that could lead to formation of water-filled channels or defects in bilayers.^109^ Together, the results show that the scaffold of TP4 is uniquely optimized for its target, allowing for superior potency and potentially higher specificity. Its unique sequence design that incorporates both AMP and CPP features is fit to insert into and, deform bilayers, translocate across membranes, and carry water across them. All these properties translate into a multifaceted mechanism of action of TP4 at cancer cell membranes, and probably, multiple modes of transfer into the cytosol. As it was argued before, the transfer can happen through various mechanisms, including endocytic pathways.^105^ Besides polyarginines, TP4 is rich in histidine, which is pH sensitive in the physiological range. Importantly, the histidine at position 3 belongs to its ATCUN motif, with the side chain contributing to coordinating metal ions (Cu^2+^, Ni^2+^).^53,110^ In future studies it will be interesting to investigate the combined roles of arginine and histidine residues in TP4’s endosomal escape for access to intracellular targets.

## Conclusion

In this study, we used biological assays to demonstrate that TP4 achieves higher efficacy than other piscidins against a set of pancreatic cancer cell lines while exhibiting selectivity for cancer cells over healthy mammalian cells. Its selectivity underscores a favorable therapeutic window. Rather than targeting protein components prone to resistance, it targets the negatively charged lipids that are enriched on the surface of cancer cells and used for immune evasion. Using a combination of structural, biophysical, and computational methods, we also investigated the molecular determinants underlying the selective anticancer activity of TP4. Mechanistically, we show that TP4 adopts a partial α-helical, yet flexible conformation upon membrane interaction and facilitates the transfer of over 100 water molecules per one molecule of TP4 molecule into the lipid bilayer. This is significantly more water than that observed previously for P1 and P3. This distinct behavior is linked to the unique C-terminal polyarginine tail of TP4, which plays a dominant role in promoting water penetration and membrane destabilization. Our results provide direct evidence that TP4 displays preferential binding to membranes enriched in negatively charged lipids such as POPS, a hallmark of cancer cell membranes. In contrast, its binding and disruptive activity are attenuated in cholesterol-rich membranes, highlighting its exquisite sensitivity to membrane composition and biophysical features. Together, these findings provide key insights into the biophysical principles governing the selectivity and activity of TP4, offering a mechanistic framework for the rational design of next-generation MAPs with enhanced specificity toward cancer cells and lower incidence of resistance.

## Supplementary Material



## Notes

IC_50_ values for all peptides tested (with and without copper) against lipogenic and nonlipogenic pancreatic cancer cell lines; IC_50_ values of an extended panel of nonpancreatic cancer and normal cell lines; correlations between expression levels and the activities of P1, P1R5, and TP4; a complete derivation of the fitting equation used for the analysis of circular dichroism data; X-ray diffraction data for all lipid compositions at 86, 93, and 97% relative humidity, including repeat spacings for all systems; neutron diffraction spectra for TP4 in bilayers with and without copper; neutron diffraction structure factors, and additional neutron reflectometry data for TP4-noR5 (PDF)

## Abbreviations

AMPs: antimicrobial peptides
ATCUN: amino-terminal copper and nickel
acetoxymethyl: Calcein AM
CD: circular dichroism
CPPs: cell penetrating peptides
DOPC: 1,2-dioleoyl-*sn*-glycero-3-phosphocholine
DOPS: 1,2-dioleoyl-*sn*-glycero-3-phosphoserine
EthD-1: Ethidium Homodimer-1
LPS: lipopolysaccharide
MAPs: membrane active peptides
MD: molecular dynamics
nMRE: normalized mean residue ellipticity
NR: neutron reflectometry
POPC: 1-palmitoyl-2-oleoyl-*sn*-glycero-3-phospho-l-choline
POPG: 1-palmitoyl-2-oleoyl-*sn*-glycero-3-phospho-l-glycerol
POPS: 1-palmitoyl-2-oleoyl-*sn*-glycero-3-phospho-l-serine
siRNA: small interfering RNA
SLD: scattering length density
SUV: small unilamellar vesicles
TP4: tilapia piscidin 4
XRD: X-ray diffraction
