# Supplementary material for: Harnessing Membrane-Active Peptides for Selective Cancer Targeting: Phosphatidylserine Recognition by Tilapia Piscidin 4
Source: JACS Au. 2026 Apr 7;6(4):2244–63. doi: 10.1021/jacsau.5c01524 (PMC13126172; doi:10.1021/jacsau.5c01524)
Supplement: Supplementary file 1 [file au5c01524_si_001.pdf]

SUPPORTING INFORMATION

Harnessing Membrane-active Peptides for Selective Cancer Targeting: Phosphatidylserine Recognition by Tilapia Piscidin 4

Wright K. Makambi[1], Soumita De[2], Dandan Li[2], Kinjal Mondal[3], Megan E. Mitchell[3,4], Navleen Kaur[1], Erik Watkins[5], Frank Heinrich[4,6], David P. Hoogerheide[4], Jeffery B. Klauda[3], Udo Rudloff[2], Myriam L. Cotten[7], Mihaela Mihailescu[1,4]

[1]Institute for Bioscience and Biotechnology Research, Rockville, MD, U.S.
[2]National Cancer Institute, Bethesda, MD, U.S.
[3]University of Maryland, College Park, MD, U.S.
[4]National Institute of Standards and Technology, Gaithersburg, MD, U.S.
[5]Oak Ridge National Laboratory, Oak Ridge, TN, U.S.
[6]Department of Physics, Carnegie Mellon University, Pittsburgh, PA, U.S.
[7]Oregon State University, Corvallis, OR, U.S.

Corresponding authors:
Mihaela Mihailescu
Institute for Bioscience and Biotechnology Research
University of Maryland
9600 Gudelsky Rd
Rockville, MD 20850
Email: ella.mihailescu@nist.gov
Phone: (240) 314-6123

Myriam L. Cotten
Department of Biochemistry and Biophysics
Oregon State University
2750 SW Campus Way
Corvallis, OR 97331
Email: cottenmy@oregonstate.edu
Phone: (541) 706-2191

Udo Rudloff
Rare Tumor Initiative, Pediatric Oncology Branch
Center for Cancer Research
National Cancer Institute
10 Center Drive – 2B 34D
Bethesda, MD 20892
Email: rudloffu@mail.nih.gov
Phone: (240) 760-6238

Table of Contents

Model Derivation……………………………………………………………………………….………………..S4

Table S1. Expression levels of relevant transmembrane proteins that control the movement of phospholipids……………………………………………………………………………………......S5

Table S2. Neutron diffraction structure factors F(h) and standard deviations of all observed Bragg diffraction peaks (h).……………………………………………………………………….…………S6

Table S3. IC_{50} values for all peptides with or without copper, against lipogenic and non-lipogenic pancreatic cancer cell lines……………………..……………………...……………………………….S7

Table S4. Q-values comparing IC_{50} values for P1 and P1R5 against lipogenic and non-lipognic pancreatic cancer cells…...……………………..………………….....………………………………..……S8

Table S5. IC_{50} values for lipogenic and non-lipogenic pancreatic cancer cell control experiment. …..……………………..……………………...…………………………………………..……………..S8

Table S6. IC_{50} values of TP4 against healthy and cancer cell lines.……...……………….……..S8

Table S7. Repeat distance for various oriented lipid bilayers in the absence and presence of TP4 …………………….……………………………………………………………………………...…...S9

Table S8. Relevant structural parameters of the neutron reflectometry models ….………………..S9

Figure S1. Anticancer activity of piscidin peptides with or without copper against lipogenic and non-lipogenic cancer cells…..……………………………………………………………………….…..S10

Figure S2. Lipogenic PANC-1 and non-lipogenic PSN-1 pancreatic cancer cells have different metabolic vulnerabilities……………………...………………………………….……………………S11

Figure S3. TP4 reduces the active mitochondrial mass in PSN-1 cancer cells. ………………..S12

Figure S4. Anticancer activity of TP4 against healthy and cancer cell lines.…………………...…S12

Figure S5. Expression levels of transmembrane proteins that control movement of phospholipids correlate with activity for P1, TP4, and P1R5……………………………………………………S13

Figure S6. X-ray diffraction data of TP4 with various mixed lipid bilayers……….……………..…...S14

Figure S7. Neutron reflectometry data for DOPC/DOPS (3:1) lipid bilayers adsorbed on silicon wafers and subsequently exposed to A) TP4 or B) TP4-noR5………………… ………….……...S15

Figure S8. Volume occupancy distributions representing the structure of the bilayer as observed by neutron reflectometry…………………………………………………………………………...S16

Figure S9. Neutron diffraction data and bilayer and water profiles for TP4 in POPC multilayers, hydrated from the vapor phase ……………………………………………………………………...S17

Figure S10. Molecular dynamics simulation results for the membrane system ……….…………S18

Model derivation

To quantify the binding affinity of peptides to lipid membranes, we fit the experimental data to a modified Langmuir binding model. From this fit, we extracted the dissociation constant (K_D), number of lipids bound per peptide (n), and maximum normalized mean residue ellipticity ($nMRE_{max}$).

At equilibrium, the dissociation constant can be defined as follows,

$$K_D = \frac{C_L * C_P}{C_{LB}} \quad (1)$$

Where C_L is the free lipid concentration, C_P is the free peptide concentration, and C_{LB} is the concentration of lipids bound to peptides.

The fraction of peptide bound (f_{PB}) and the fraction of lipid bound(f_{LB}), can be defined as follows:

$$f_{PB} = \frac{C_{PB}}{C_{Pt}} \quad (2)$$

$$f_{LB} = \frac{nC_{PB}}{C_{Lt}} \quad (3)$$

$$f_{LB} = \frac{C_{LB}}{C_L + C_{LB}} \quad (4)$$

Here, C_{PB} is the concentration of peptide bound to lipids, C_{Pt} is the total peptide concentration, C_{Lt} is the total lipid concentration, and n is the number of lipids bound to a peptide.

By substituting the definition of K_D into equation 4, we obtain the classical Langmuir isotherm.

$$f_{LB} = \frac{C_P}{K_D + C_P} \quad (5)$$

Combining equations (3) and (5), we can derive the expression for C_{PB}:

$$C_{PB} = \frac{C_{Lt} * C_P}{n\,(K_D + C_P)} \quad (6)$$

Substituting this into the definition of *equation 2* gives:

$$f_{PB} = \frac{1}{C_{Pt}} * \frac{C_{Lt} * C_P}{n*(K_D + C_P)} \quad (7)$$

Rearranging this expression yields:

$$f_{PB} * n \left(\frac{K_D}{C_P} + 1\right) = \frac{C_{Lt}}{C_{Pt}} \quad (8)$$

or, expressed in terms of f_{PB} and C_{Ptotal}:

$$f_{PB} * n \left(\frac{K_D}{C_{Pt}(1 - f_{PB})} + 1\right) C_{Pt} = C_{Lt} \quad (9)$$

Finally, the fraction of bound peptide can be related to normalized mean residue ellipticity measurements:

$$nMRE = f_{PB} * (nMRE_{max} - nMRE_{min}) + nMRE_{min} \quad (10)$$

Where *nMRE* is the measured normalized MRE, $nMRE_{min}$, is the minimum normalized MRE (set to 1), and nMR_{max} is the maximum value observed for a given peptide-lipid mixture.

Supplementary Tables

Table S1. Expression levels of relevant transmembrane proteins that control the movement of phospholipids.

Enzyme class	Enzyme	ACCESSION	YAPC	PANC1	PSN1	PK59	PK8	MiaPaca2
Flippase	ATP11A	BF439472	6.31	5.82	5.66	6.95	4.45	4.96
	ATP11A	AK024264	6.07	6.51	5.74	6.59	5.59	5.33
	ATP11A	AL161996	7.29	6.85	6.28	7.29	4.68	6.10
	ATP11A	AW068936	10.07	9.41	8.75	10.35	6.71	8.38
	ATP11A	BE745453	4.34	4.74	4.31	4.27	3.98	4.67
	ATP11A	BE745453	4.23	4.32	4.05	4.02	4.00	4.30
	ATP11B	BC033880	6.85	7.52	6.49	6.35	6.99	6.58
	ATP11B	BC033880	6.90	7.87	6.05	6.09	6.73	6.29
	ATP11B	AK093727	6.25	6.80	6.02	5.71	6.47	6.24
	ATP11B	AK093727	8.11	8.51	7.86	7.09	7.79	7.83
	ATP11B	AB023173	8.67	9.34	8.18	8.43	8.07	8.36
	ATP11B	AA639797	4.74	5.75	4.52	4.48	4.48	4.82
	ATP8A1	BC020943	3.37	3.36	3.18	3.35	3.34	3.11
	ATP8A1	AB013452	4.30	4.37	4.22	4.23	4.42	4.28
	ATP8A1	AI769688	3.36	3.34	4.69	4.02	3.78	4.04
	ATP8A2	AU146927	4.65	4.59	4.51	5.69	4.65	4.32
	ATP8A2	NM_016529	4.48	4.26	4.34	4.98	4.30	4.26
	ATP8A2	BE504186	5.06	4.78	4.95	5.39	4.98	4.96
Scramblase	ANO1	BC033036	7.52	6.01	6.43	7.92	7.43	5.88
	ANO1	NM_018043	9.71	3.36	5.64	10.08	9.30	3.86
	ANO6	AL041280	9.42	10.72	9.57	9.85	9.60	9.07
	ANO6	N36984	4.77	5.56	4.99	5.03	4.98	4.48

Table S2. Neutron diffraction structure factors F(h) and standard deviations (1 SD) of all observed Bragg diffraction peaks (h). Oriented multilayers of TP4/POPC 1:25 molar ratio were measured in H_2O at 93% relative humidity and 23 °C. Structure factors for the same sample of TP4/POPC hydrated in 20% ^2H_2O (^2H_2O:H_2O=1:4) and an equivalent sample that contain D7-PC (D7-PC:POPC=1:5), measured under the same conditions of hydration and temperature.

	H_2O		^2H_2O:H_2O (1:4)		D_7PC:POPC (1:5)	
**h**	**F(h)**	**S.D.**	**F(h)**	**S.D.**	**ΔF(h)**	**S.D.**
1	-1.391	0.031	-10.061	0.014	0.376	0.028
2	-7.042	0.030	-3.943	0.024	-6.352	0.018
3	2.372	0.077	2.137	0.059	2.492	0.039
4	-0.980	0.247	-0.980	0.192	-0.760	0.189
5	-1.361	0.328	-1.361	0.163	-1.544	0.134

Table S3. ***IC_{50}*** **values for all peptides with or without copper, against lipogenic and non-lipogenic pancreatic cancer cell lines.**
IC_{50} are reported with 95% confidence intervals.

IC50 (µmol/L)	Cell line	P1	P1-Cu	P1R5	P1R5-Cu	TP4	TP4-Cu	P3	P3-Cu
Lipogenic	**PK-59**	4.23 (3.83-4.71)	1.92 (1.68-2.19)	2.45 (2.32-2.57)	1.34 (1.25-1.45)	1.42 (1.38-1.47)	1.19 (1.12-1.28)	ND[a] (ND)[a]	5.33 (4.51-6.38)
	**PK-8**	5.60 (4.58-6.95)	2.28 (2.07-2.51)	3.19 (2.72-3.86)	3.17 (2.80-3.65)	1.97 (1.86-2.10)	1.86 (1.75-1.99)	36.38 (ND)[a]	5.02 (4.38-5.80)
	**PANC-1**	8.37 (7.42-9.59)	5.86 (5.44-6.31)	7.66 (7.10-8.34)	5.94 (3.95-13.35)	2.59 (2.44-2.75)	2.47 (2.27-2.70)	~50.1 (ND)[a]	8.62 (7.77-9.79)
Non-lipogenic	**PSN-1**	4.77 (4.51-5.04)	2.29 (2.12-2.46)	3.17 (2.99-3.38)	1.99 (1.77-2.26)	1.74 (1.66-1.83)	1.82 (1.73-1.93)	ND[a] (ND)[a]	5.32 (4.75-5.95)
	**MIA PaCa-2**	6.69 (6.14-7.27)	4.70 (4.39-5.04)	6.03 (5.18-7.12)	4.24 (3.73-4.94)	2.10 (2.00-2.20)	2.42 (2.22-2.64)	14.94 (11.18-35.51)	6.65 (5.90-7.47)
	**YAPC**	5.92 (5.56-6.32)	2.90 (2.73-3.09)	4.03 (3.72-4.40)	2.65 (2.39-2.99)	2.02 (1.89-2.17)	2.22 (1.82-2.63)	ND[a] (ND)[a]	5.25 (4.59-6.06)

[a]ND: Not determined

Table S4. *Q*-values comparing IC_{50} values for P1 and P1R5 against lipogenic and non-lipogenic pancreatic cancer cells.

		Q-values[a]
		P1 vs P1R5
**Lipogenic**	PK-59	0.23
	PK-8	0.40
	PANC1	0.86
**Non-lipogenic**	PSN1	0.22
	MIAPaCa2	0.37
	YAPC	0.22

[a] *Q*-values from a paired t-test with FDR = 0.01 and comparison to P1.

Table S5. IC_{50} values for lipogenic and non-lipogenic pancreatic cancer cell control experiment. IC_{50} values are reported with 95% confidence intervals.

IC_{50} (μmol/L)	PSN-1	PANC-1
**R-GNE-140[a]**	5.12 (4.54-5.79)	25.15 (15.0-200)
**GSK1940029[b]**	32.36 (ND)[c]	(ND)[c]

[a] R-GNE-140; lactate dehydrogenase A (LDHA) inhibitor
[b] GSK1940029; stearoyl-CoA desaturase (SCD) inhibitor
[c] ND: not determined

Table S6. IC_{50} values of TP4 against healthy and cancer cell lines. IC50 are reported with 95% confidence intervals.

Cell line	Hela	PC3M	GM05659	HEK293	HTT Fibroblast
IC_{50} (μmol/L)	7.04 (6.7-8.15)	5.32 (4.92-5.73)	ND[a]	ND[a]	ND[a]

[a] ND: not determined

Table S7. **Repeat distance for various oriented lipid bilayers in the absence and presence of TP4.**

Lipid	Hydration	Peptide	Repeat distance (Å)	Δ Repeat distance (Å)[a,b]
POPC	86	None	51.91	-0.1
		TP4	51.81	
	93	None	52.42	0.38
		TP4	52.80	
	97	None	53.53	0.40
		TP4	53.93	
POPC/POPS (5:1)	86	None	52.48	0.89
		TP4	53.37	
	93	None	52.85	5.39
		TP4	58.24	
	97	None	53.77	21.21
		TP4	74.98	
POPC/Cholesterol (2:1)	86	None	55.09	-2.63
		TP4	52.46	
	93	None	55.82	-1.99
		TP4	53.83	
	97	None	57.36	-1.19
		TP4	56.17	
POPC/POPS/Cholesterol (5:1:2.5)	86	None	54.86	0.66 / 1.31
		TP4	55.5 / 50.7	
	93	None	56.13	1.6 / -3.11
		TP4	57.7 / 53.0	
	97	None	57.04	1.56 / -1.77
		TP4	58.6 / 55.3	

Table S8. Relevant structural parameters of the neutron reflectometry models.

Peptide	WT-TP4	TP4-noR5
Area per lipid (outer leaflet) ($Å^2$)	$73.3^{+1.3}_{-1.3}$	$76.4^{+1.5}_{-1.5}$
Membrane hydrophobic thickness (Å)	$30.44^{+0.07}_{-0.08}$	$30.55^{+0.07}_{-0.05}$
Submembrane thickness (no peptide) (Å)	$0.83^{+0.14}_{-0.14}$	$0.94^{+0.06}_{-0.10}$
Submembrane thickness (with peptide) (Å)	$3.56^{+0.42}_{-0.42}$	$1.30^{+0.90}_{-0.54}$
Change in acyl chain thickness with peptide (per leaflet) ($Å^2$)	$-1.84^{+0.13}_{-0.12}$	$-1.54^{+0.11}_{-0.39}$

Supplementary Figures

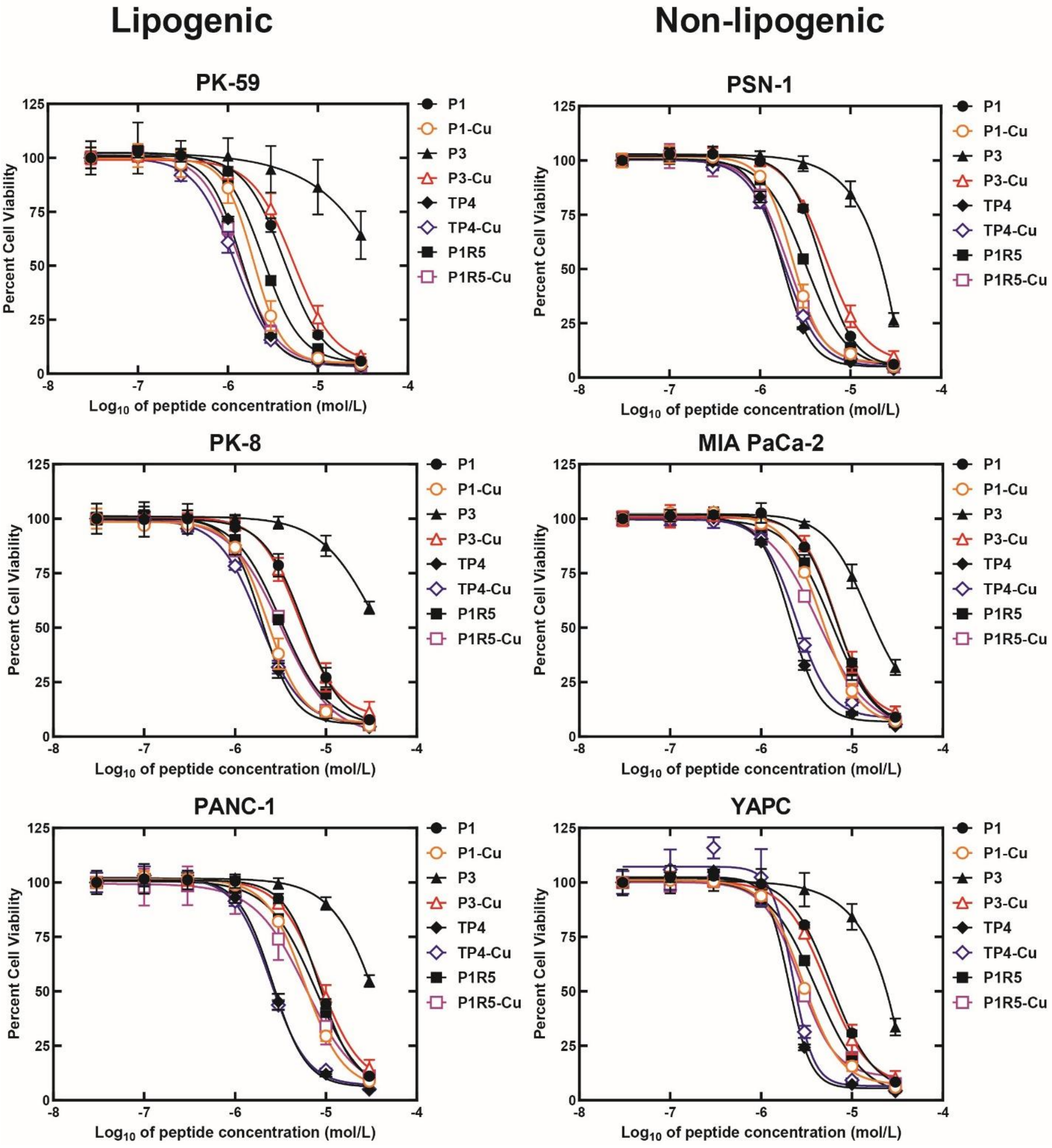

Figure S1. Anticancer activity of piscidin peptides with or without copper against lipogenic and non-lipogenic cancer cells. After peptides (P1, P3, TP4, or P1R5) were mixed with cells to the final concentrations indicated, the mixture was incubated at 37 °C for 24 hours. Cell growth was assessed using the CellTiter-Glo assay, with levels of untreated cells normalized to 100%. Metallation was done in a 1:1 molar ratio between the peptide and metal using aqueous $CuCl_2$ (Hampton Research, Aliso Viejo, CA). The pH was carefully adjusted to 7.4 since protons are released by the peptide upon metal binding.[1]

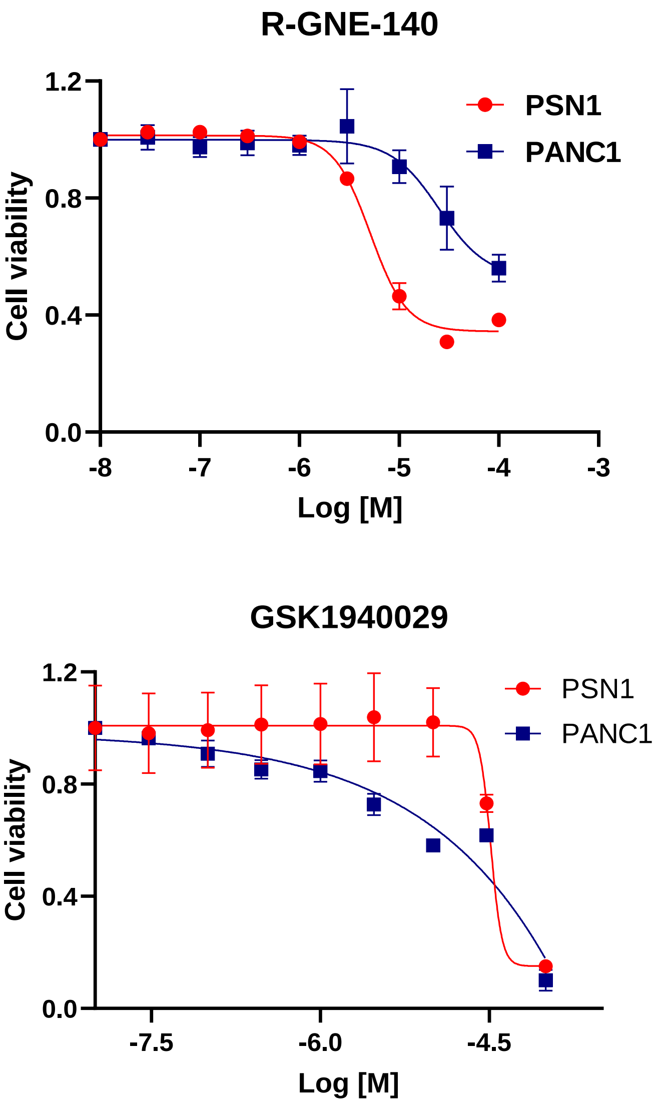

Figure S2. Lipogenic PANC-1 and non-lipogenic PSN-1 pancreatic cancer cells have different metabolic vulnerabilities. PANC1 (dose response in blue) and PSN1 cells (dose response in red) were treated with vehicle or by (R)-GNE-140, a lactate dehydrogenase A (LDHA) inhibitor or GSK1940029, a stearoyl-coa desaturase (SCD) inhibitor for 72 h. After treatment cell viability was measured by CellGiter-Glo assay, with levels of untreated cells normalized to 100%. IC_{50} values are listed in Table S5. Error bars represent the standard deviation for n = 2 replicates.

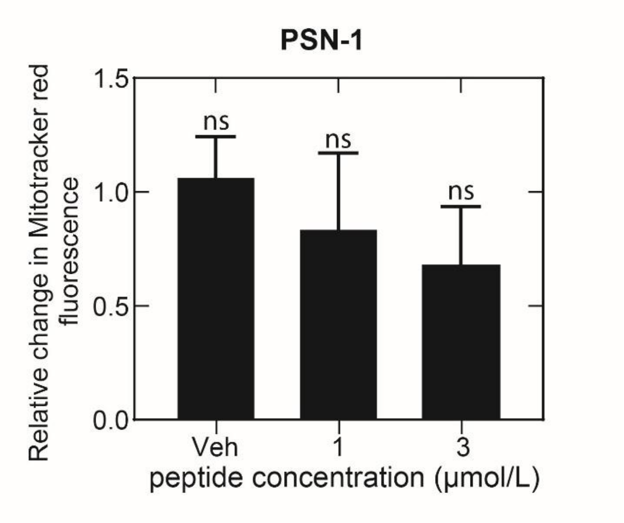

Figure S3. Effect of TP4 on the active mitochondrial mass in PSN-1 cancer cells. PSN-1 cells were exposed to TP4 at indicated concentrations followed by MitoTracker staining and quantification of total emitted fluorescence. Total fluorescence of vehicle control was normalized to 100%. Error bars represent the standard deviation for n = 2 replicates.

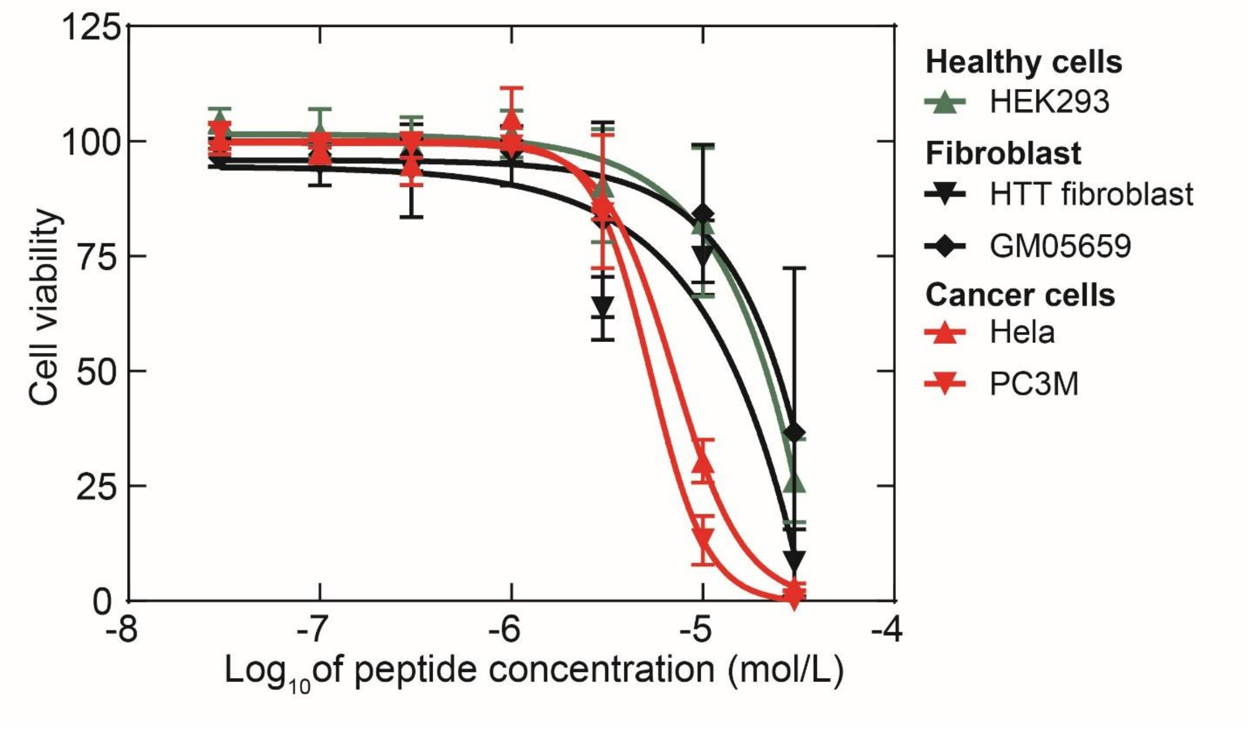

Figure S4. Anticancer activity of TP4 against healthy and cancer cell lines. Cancer cells (Hela PC3M), Fibroblast (HTT fibroblast and GM05659), and healthy epithelial cells (HEK293) were exposed to TP4 at the indicated concentration. Mixtures were incubated at 37 °C for 24 hours. Following

incubation, cell growth was assessed using the CellTiter-Glo assay with levels of untreated cells normalized to 100%. Error bars represent the standard deviation for $n = 2$ replicates.

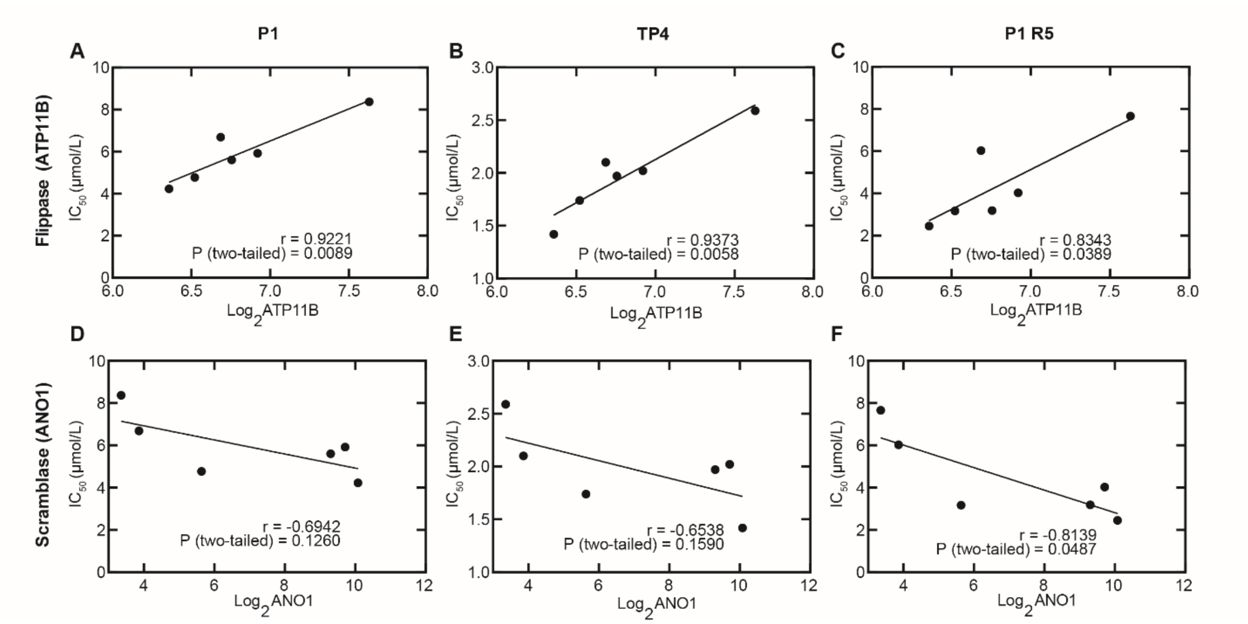

Figure S5. Expression levels of transmembrane proteins that control movement of phospholipids correlate with activity for P1, TP4, and P1R5. Gene expression levels indicate HumanHT-12 v4 expression BeadChip array signals, which were normalized to expression levels of housekeeping genes and log2-transformed. The correlation coefficient (r) from the Pearson correlation coefficient test is indicated.

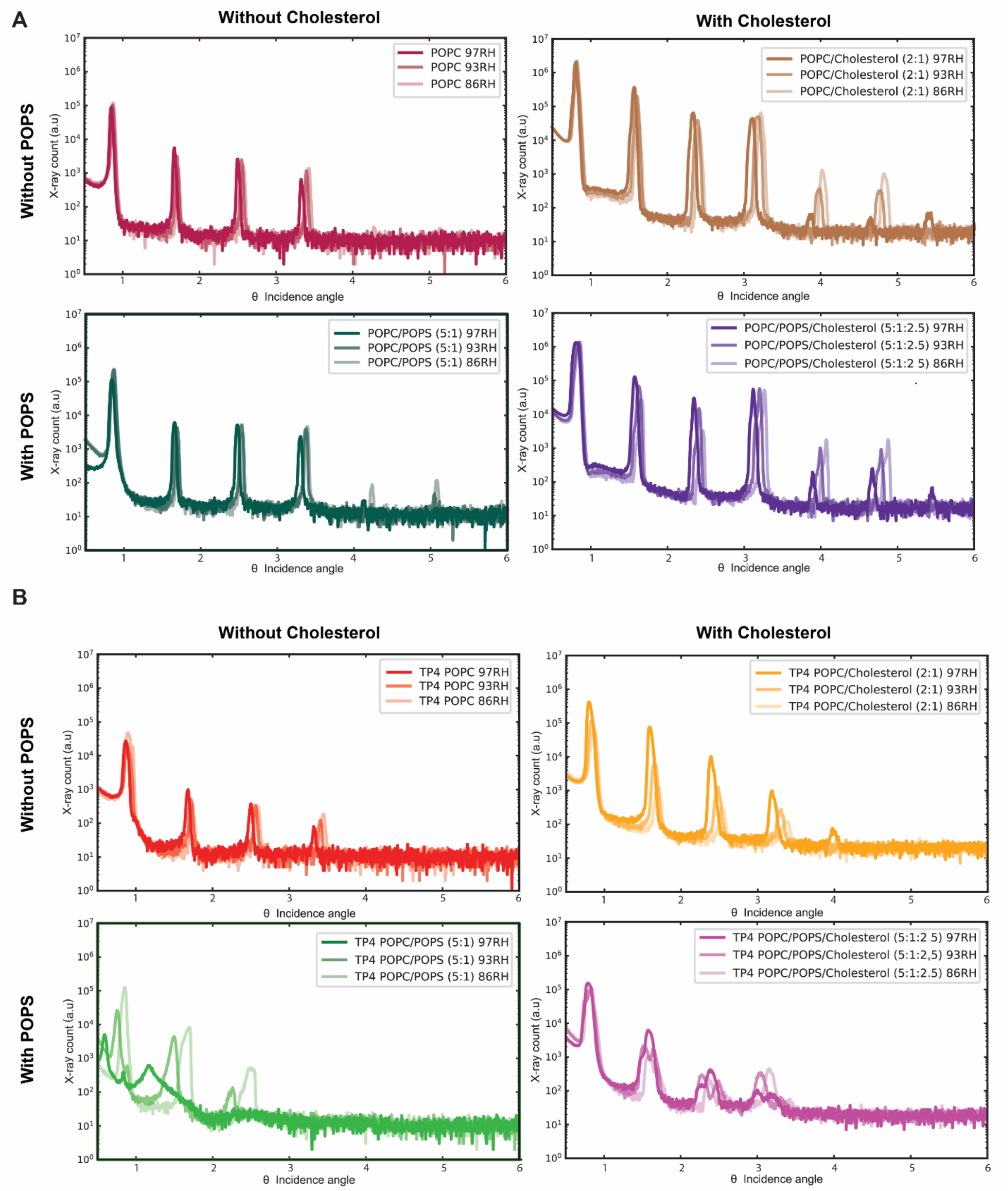

Figure S6. **X-ray diffraction data of TP4 with various mixed lipid bilayers.** Bragg diffraction from oriented lamellar samples **A)** without and **B)** with TP4. All samples were produced at a P/L of 1:25 and were measured at 86%, 93%, or 97% relative humidity and 25 °C. Structural changes in the membrane bilayer as observed by X-ray diffraction.

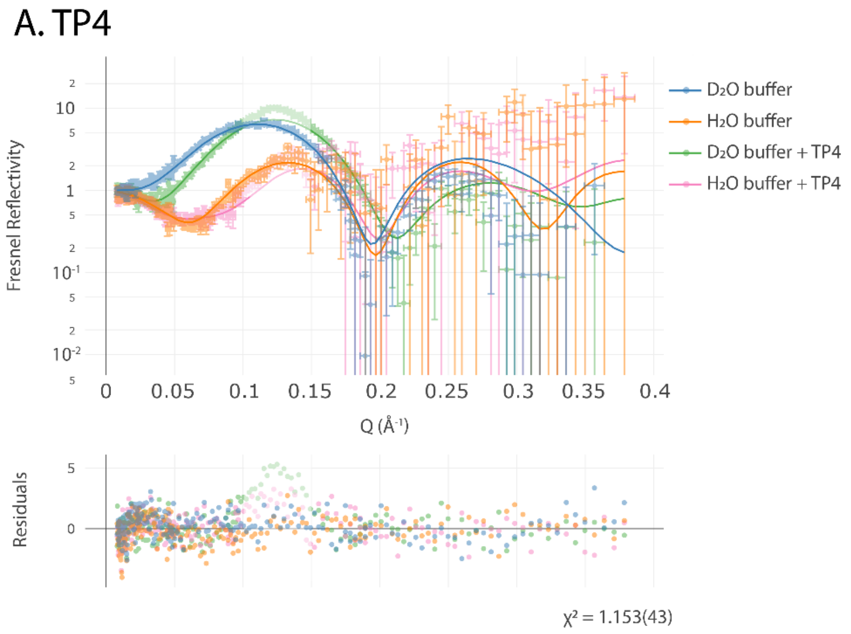

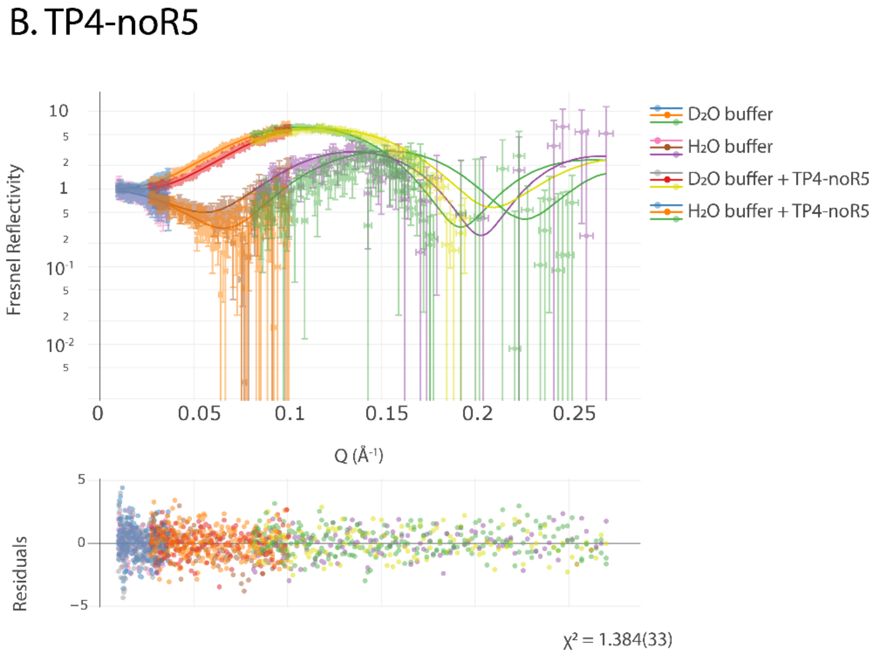

Figure S7. Neutron reflectometry data for DOPC/DOPS (3:1) lipid bilayers adsorbed on silicon wafers and subsequently exposed to A) TP4 or B) TP4-noR5. Data is normalized to the Fresnel reflectivity of the silicon/buffer interface. Uncertainties represent 68% confidence intervals derived from the neutron counting uncertainties. Curves are reflectivity patterns optimized to the models described in the main text. The multilayer peaks were removed from the analysis (shading), and the optimized reflectivity patterns were obtained without these data. Goodness of fit metrics were **A)** χ^2=1.153 and **B)** χ^2=1.384 for TP4 and TP4-noR5, respectively.

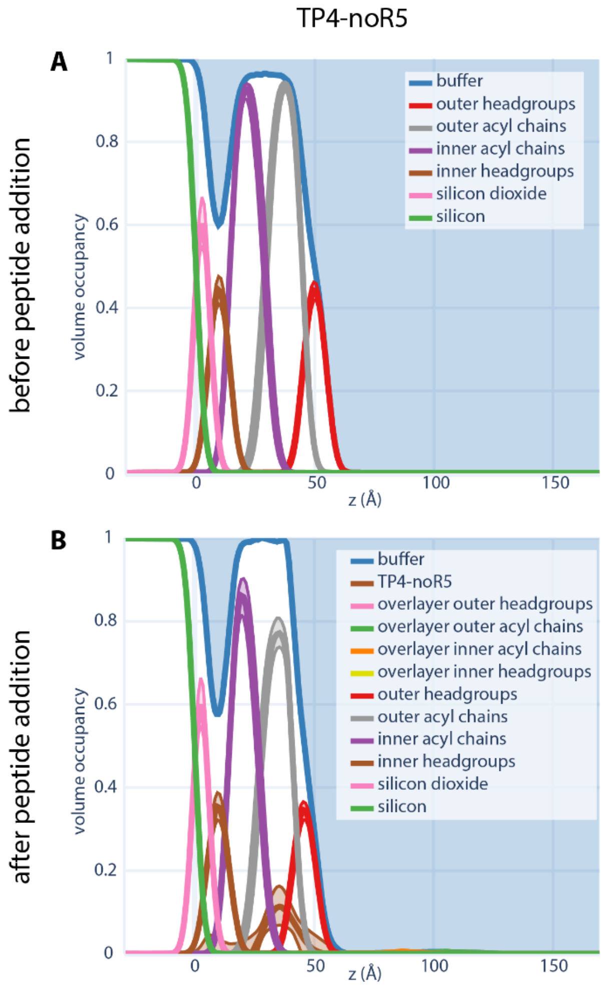

Figure S8. Volume occupancy distributions representing the structure of the bilayer as observed by neutron reflectometry. DOPC/DOPS (3:1) vesicles were allowed to adsorb onto a silicon wafer until fully covered. TP4-noR5 was injected using a syringe pump. Free lipids and TP4-noR5 were washed off the wafer between each step. Results were collected **A)** Before and **B)** After adding TP4-noR5.

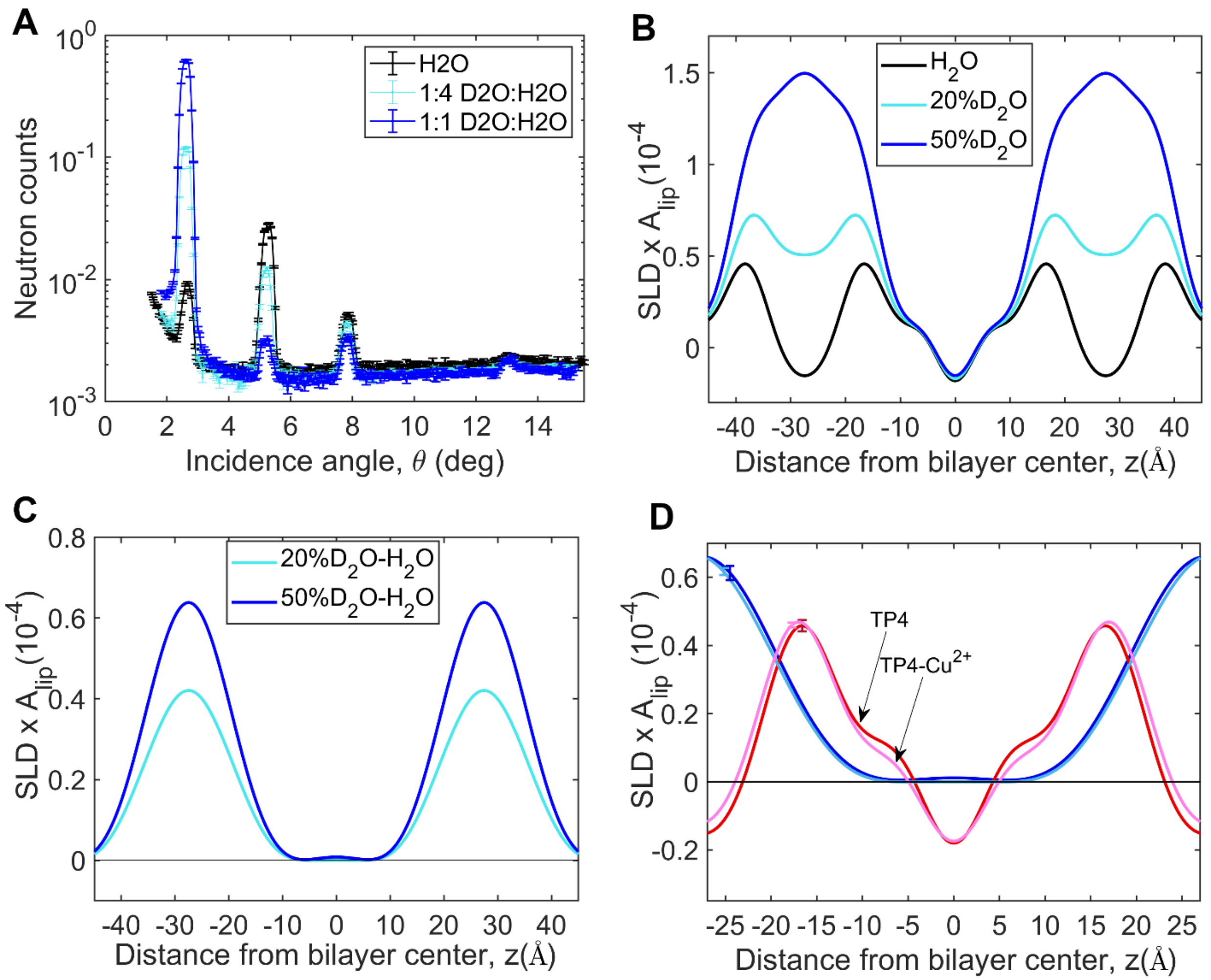

Figure S9. Neutron diffraction data and bilayer and water profiles for TP4 in POPC multilayers, hydrated from the vapor phase. A) Neutron diffraction data from lipid multilayers of TP4/POPC 1:25, measured at 93%relative humidity and 23 °C and hydrated with increasing amounts of deuterated water (D2O). **B)** Scattering length density (SLD) profiles for POPC bilayers with TP4 calculated from the data in A). The graph shows one full bilayer centered at z = 0 and part of the adjacent bilayer profiles. **C)** Water profiles in POPC bilayers with TP4 determined by subtracting the H_2O bilayer profile from the D_2O profiles in B). **D)** A POPC bilayer containing TP4 (dark red) and its corresponding water distribution (dark blue) and a POPC bilayer with TP4-Cu^{2+} (pink) and its water distribution (light blue). The repeat distances (d) were 54.9 Å (± 0.1 Å) for TP4 and 56.4 Å (± 0.1 Å) for TP4-Cu^{2+}. The water accumulates mainly in between adjacent bilayers, between the phospholipid headgroups but it extends into the bilayer interior, pulled alongside the peptide. No significant difference in bilayer and water SLD profiles are observed between TP4 and TP4-Cu^{2+}. Error bars illustrate the magnitude of the uncertainties (± 1 standard deviation) in the profiles.

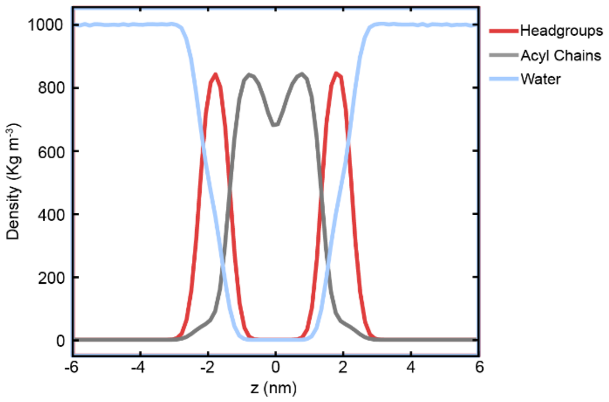

Figure S10. Molecular dynamics simulation results for the membrane system. Electron density profiles of the various groups located along the POPC/POPS (5:1) lipid bilayer.

References

(1) Rai, R. K.; De Angelis, A.; Greenwood, A.; Opella, S. J.; Cotten, M. L. Metal-ion Binding to Host Defense Peptide Piscidin 3 Observed in Phospholipid Bilayers by Magic Angle Spinning Solid-state NMR. *Chemphyschem : a European journal of chemical physics and physical chemistry* **2018**.
